# Supplementary material for: Deep Learning‐Assisted Design of Novel Promoters in Escherichia coli
Source: Adv Genet (Hoboken). 2023 Nov 15;4(4):2300184. doi: 10.1002/ggn2.202300184 (PMC10716054; doi:10.1002/ggn2.202300184)
Supplement: Supplementary file 1 — Supporting Information [file GGN2-4-2300184-s004.pdf]

ADVANCED GENETICS
Open Access

Supporting Information

for *Advanced Genetics*, DOI 10.1002/ggn2.202300184

Deep Learning-Assisted Design of Novel Promoters in *Escherichia coli*

*Xinglong Wang, Kangjie Xu, Yameng Tan, Shangyang Yu, Xinyi Zhao and Jingwen Zhou**

Supplementary File

Deep learning-assisted design of novel promoters in *Escherichia coli*

Xinglong Wang[1,2], Kangjie Xu[1,2], Yameng Tan[1,2], Shangyang Yu[1,2],Xinyi Zhao[1,2], Jingwen Zhou[1,2,3,*]

[1] Engineering Research Center of Ministry of Education on Food Synthetic Biotechnology and School of Biotechnology, Jiangnan University, 1800 Lihu Road, Wuxi, Jiangsu 214122, China;

[2] Science Center for Future Foods, Jiangnan University, 1800 Lihu Road, Wuxi, Jiangsu 214122, China;

[3] Jiangsu Province Engineering Research Center of Food Synthetic Biotechnology, Jiangnan University, Wuxi 214122, China.

* Corresponding author: Jingwen Zhou.

Science Center for Future Foods, Jiangnan University, 1800 Lihu Road, Wuxi, Jiangsu 214122, China.

Phone: +86-510-85914371, Fax: +86-510-85914371.

E-mail: zhoujw1982@jiangnan.edu.cn.

Supplementary Figures

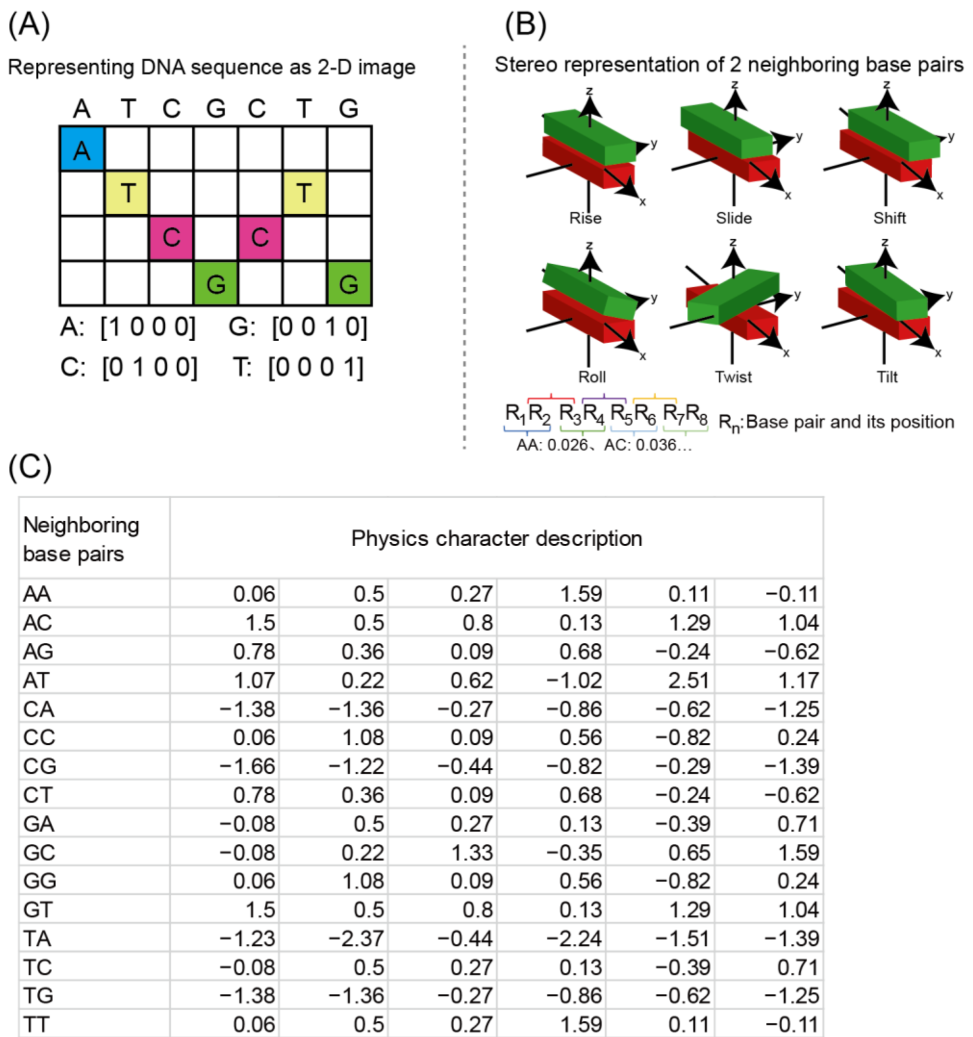

(C)

Neighboring base pairs	Physics character description					
AA	0.06	0.5	0.27	1.59	0.11	−0.11
AC	1.5	0.5	0.8	0.13	1.29	1.04
AG	0.78	0.36	0.09	0.68	−0.24	−0.62
AT	1.07	0.22	0.62	−1.02	2.51	1.17
CA	−1.38	−1.36	−0.27	−0.86	−0.62	−1.25
CC	0.06	1.08	0.09	0.56	−0.82	0.24
CG	−1.66	−1.22	−0.44	−0.82	−0.29	−1.39
CT	0.78	0.36	0.09	0.68	−0.24	−0.62
GA	−0.08	0.5	0.27	0.13	−0.39	0.71
GC	−0.08	0.22	1.33	−0.35	0.65	1.59
GG	0.06	1.08	0.09	0.56	−0.82	0.24
GT	1.5	0.5	0.8	0.13	1.29	1.04
TA	−1.23	−2.37	−0.44	−2.24	−1.51	−1.39
TC	−0.08	0.5	0.27	0.13	−0.39	0.71
TG	−1.38	−1.36	−0.27	−0.86	−0.62	−1.25
TT	0.06	0.5	0.27	1.59	0.11	−0.11

Figure S1 Feature extraction methods

(A) DNA sequence feature extraction using one-hot method. (B) Sequence feature extraction using pseDNC[3]. (C) The physicochemical properties for the 16 different di-nucleotides in DNA[3].

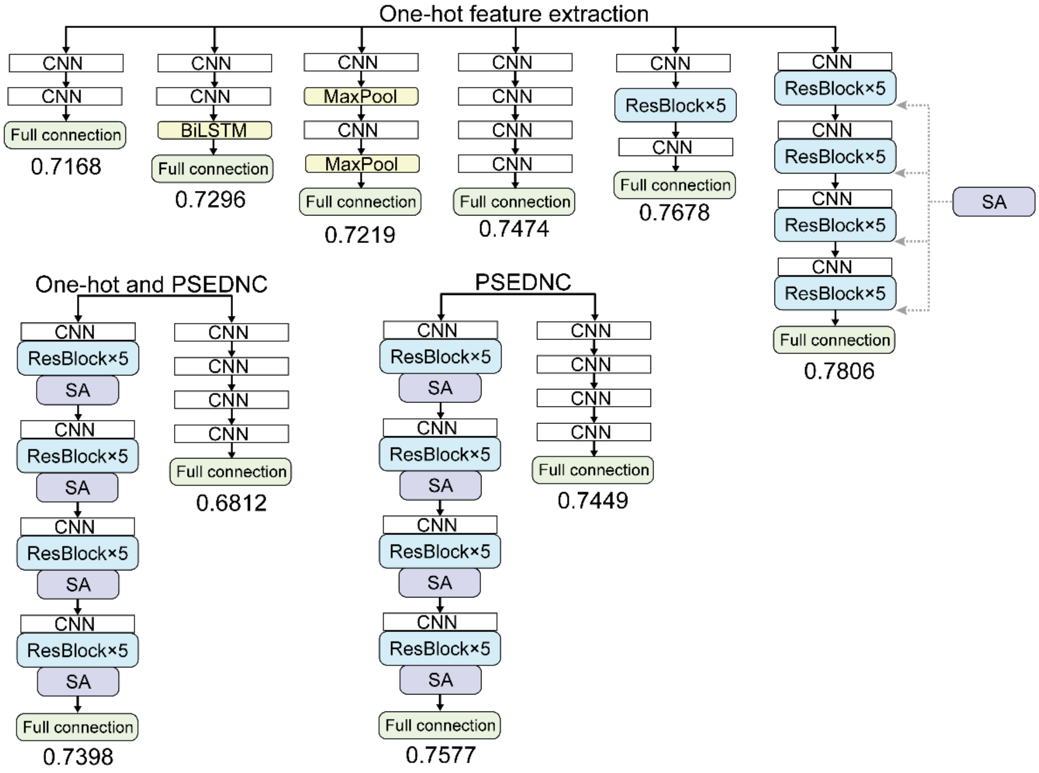

Figure S2 Optimization of deep learning model

Deep learning model PromoS architected using different blocks and their accuracy for predicting strong/weak promoters were shown. Noted CNN represented Convolutional Neural Network, BiLSTM represented Bidirectional-LSTM, and SA represented self-attention. The accuracy achieved using 10-fold cross validation.

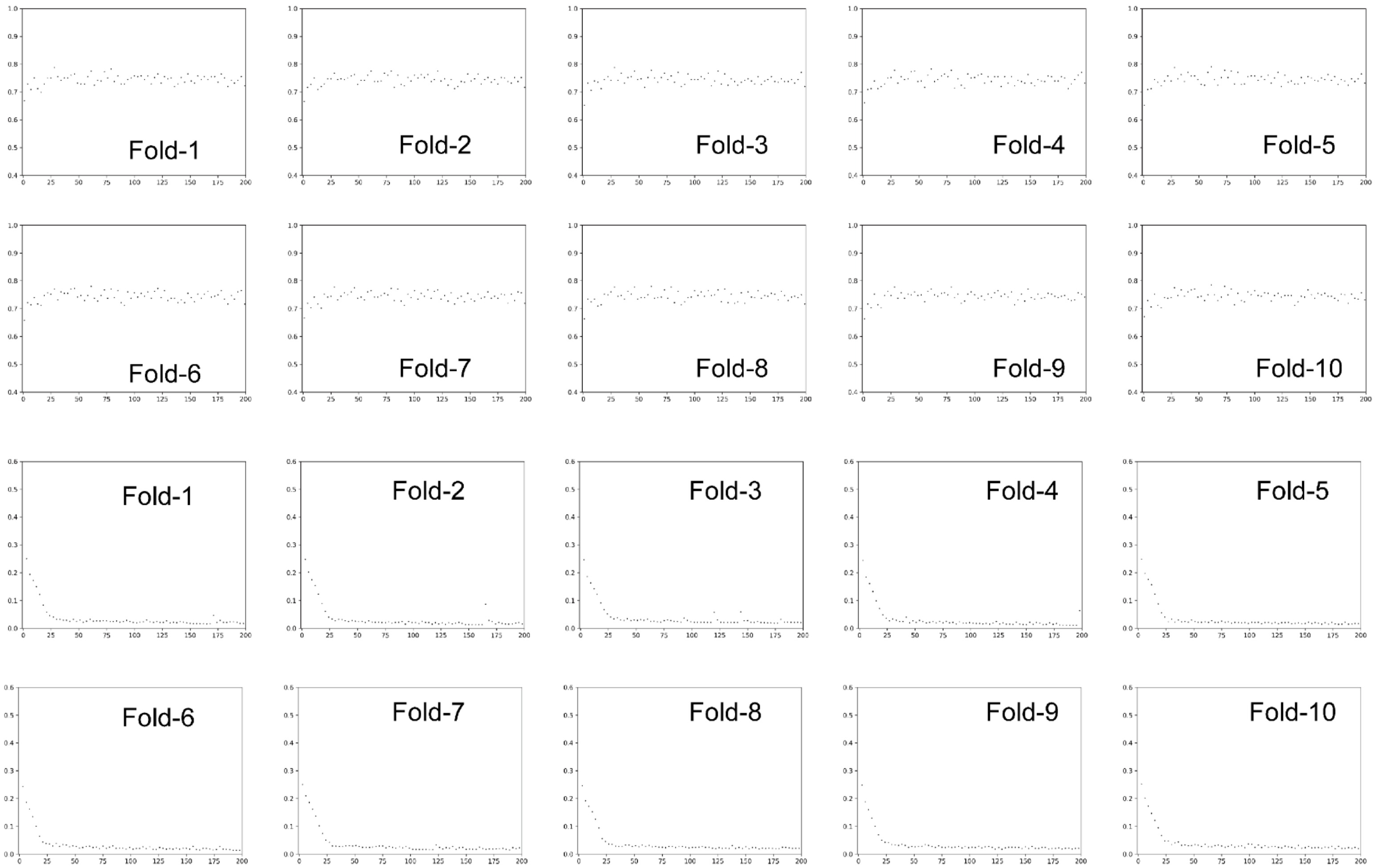

Figure S3 Training of PromoS

Training loss (bottom two lines) and accuracy (top two lines) of PromoS using 10-fold cross validation.

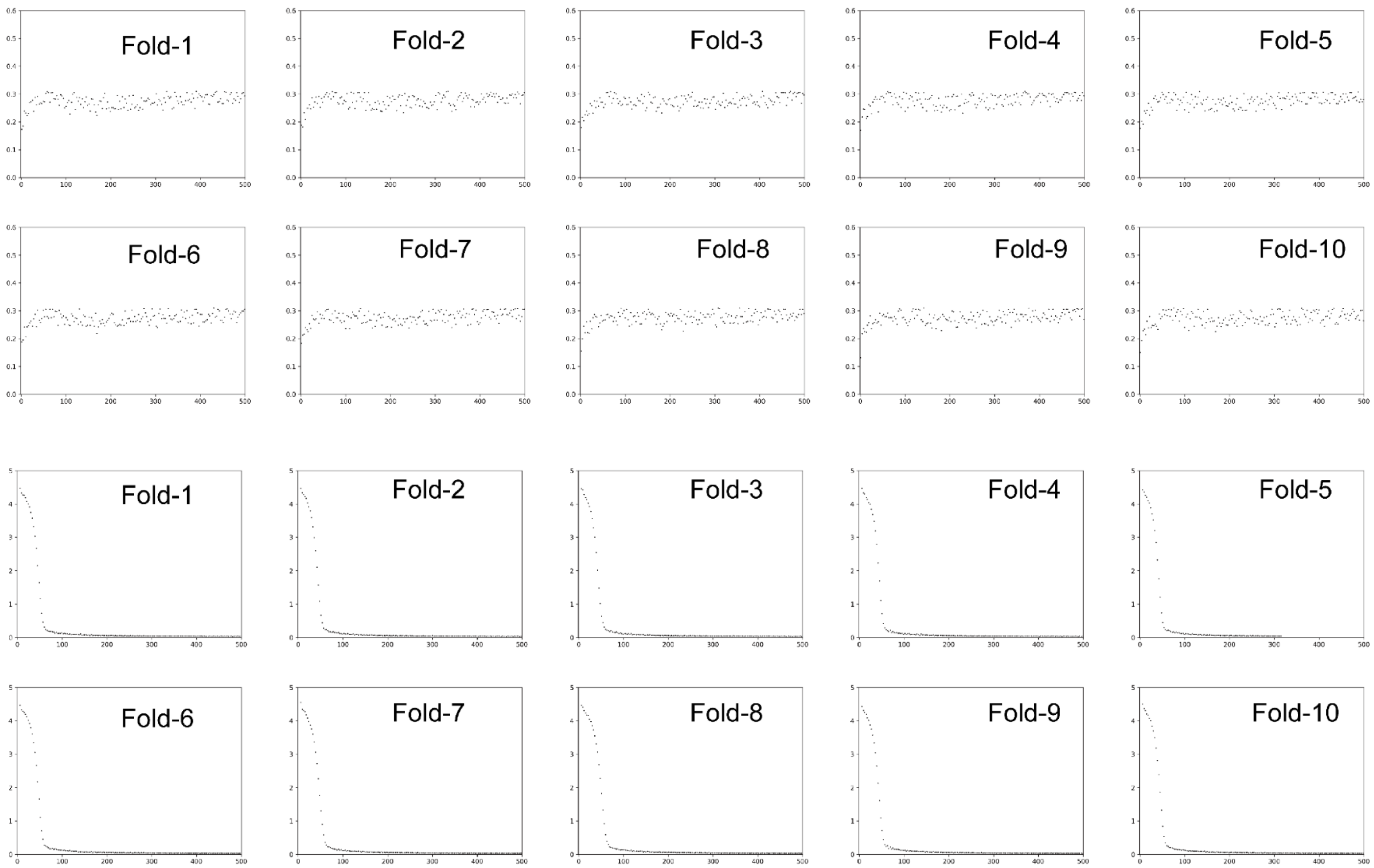

Figure S4 Training of PromoA

Training loss (bottom two lines) and accuracy (top two lines) of PromoA using 10-fold cross validation.

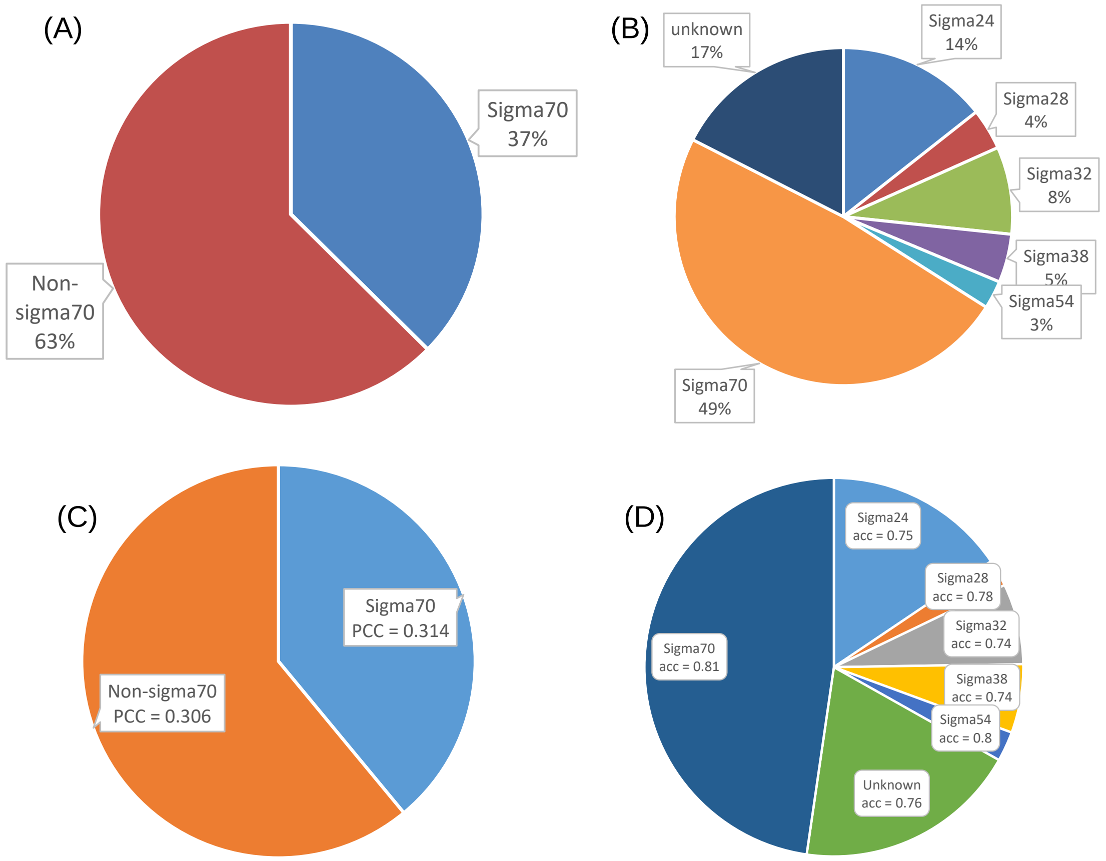

Figure S5 Classification and prediction accuracy against two datasets

The promoter classification of RegulonDB[4] (A) and NDB[2] (B). PromoA and PromoS accuracy against NDB (C) and RegulonDB (D).

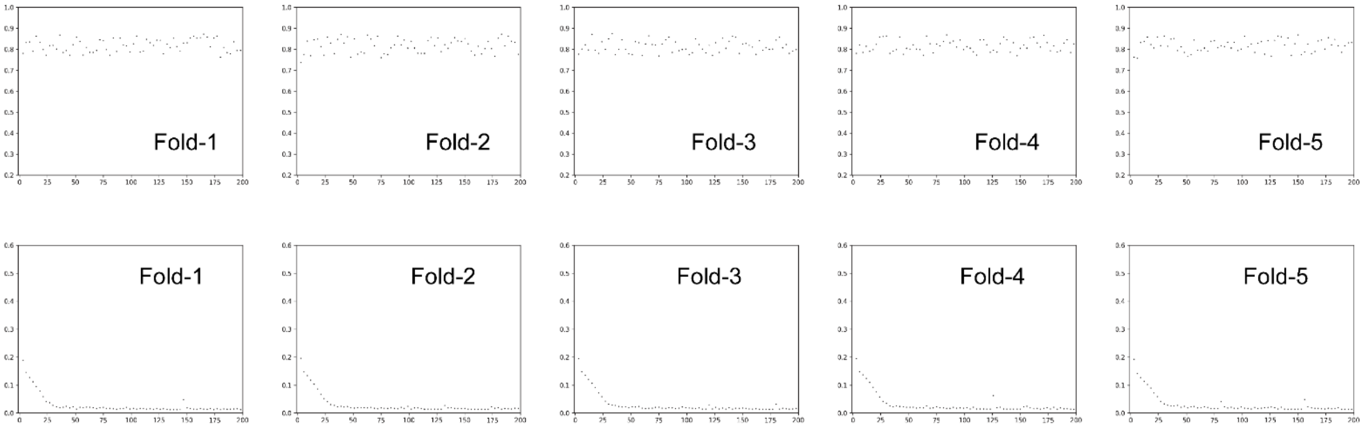

Figure S6 Training of PromoR

Training loss and accuracy of PromoR using RegulonDB or combined dataset. The combined dataset contains RegulonDB[4], NDB[2], and 11,884 non-promoters. Training loss (bottom two lines) and accuracy (top two lines) of PromoR using 10-fold cross validation.

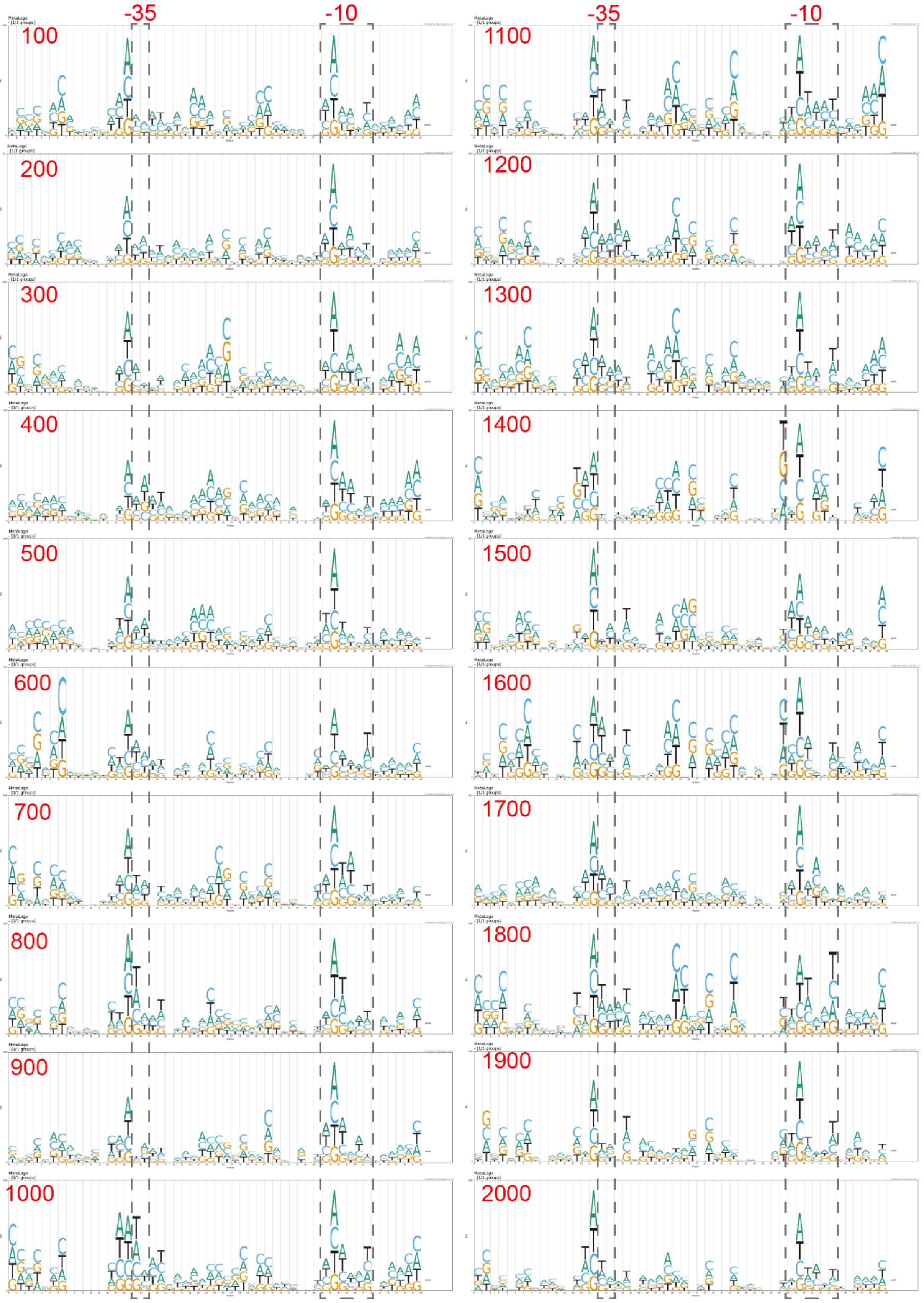

Figure S7 Sequence logo of generated promoters by PromoDiff

Promoters were generated by PromoDiff during its training, 1024 samples were generated every 100 epochs for sequence logo analysis. Sequence logo generated by Metalogo[5].

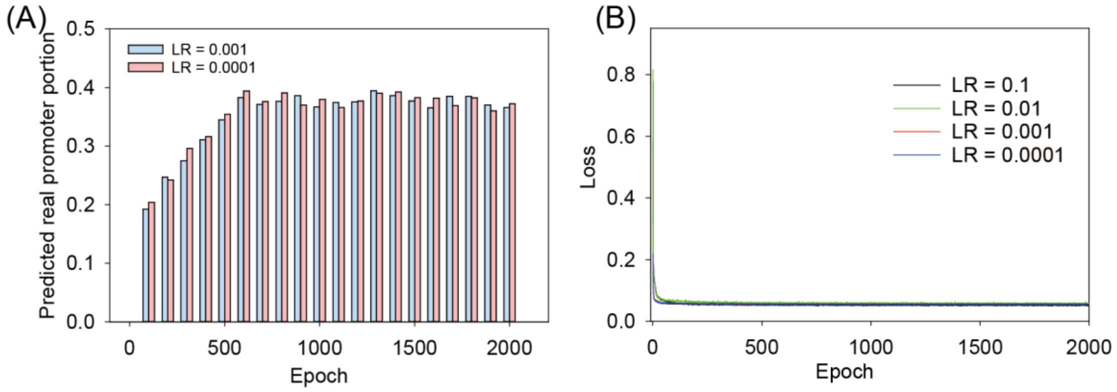

Figure S8 Characterization of the diffusion model

(A) The predicted real promoters' portion of the generated promoters by PromoDiff. (B) The loss of using different learning rate.

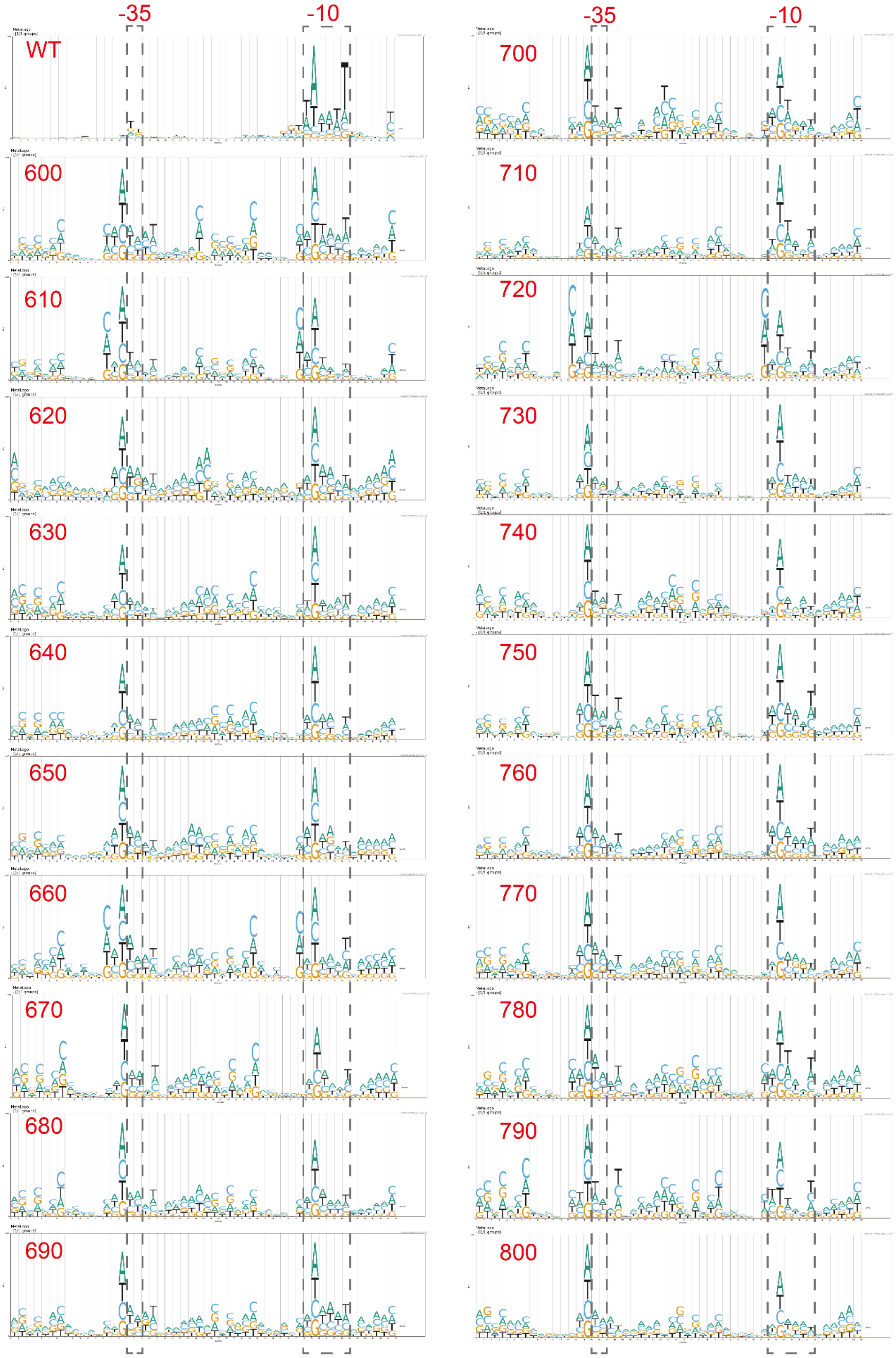

Figure S9 Sequence logo analysis of generated promoters

Promoters were generated every 10 epoch during PromoDiff training, 5000 samples were generated for sequence logo analysis[5].

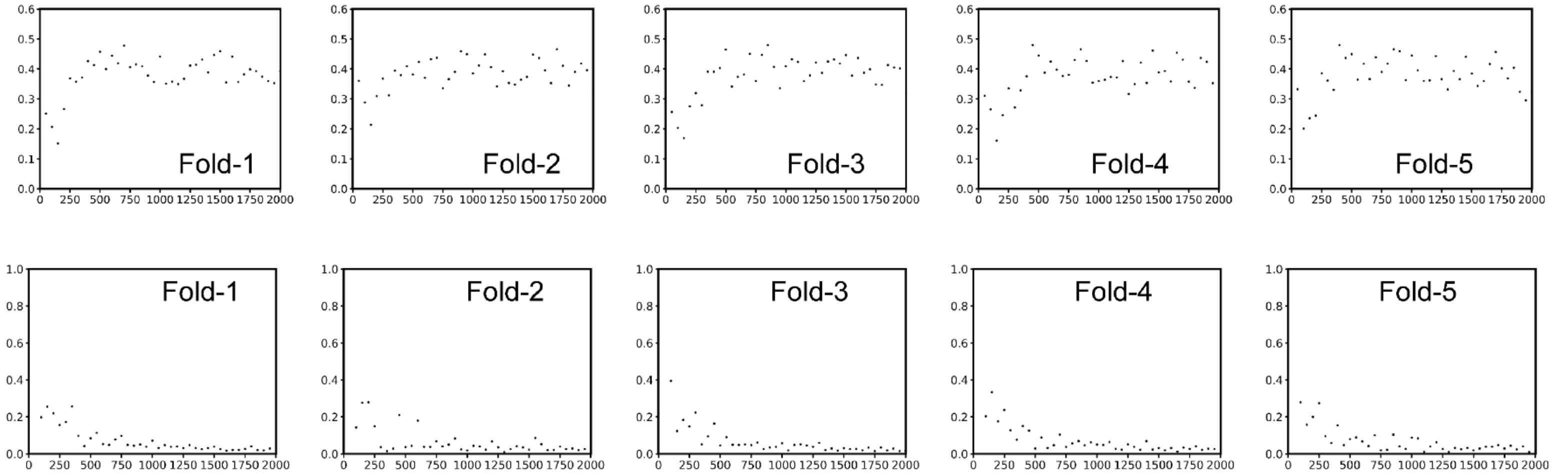

Figure S10 Training of PromoNet

Training loss (bottom two lines) and accuracy (top two lines) of PromoNet using 5-fold cross validation.

(A)

```
           NcoI
AAGGAGATATACCATGGGCAGCAGCCATCATCATCATCATCACAGCAGCGGCCTGGTGCCGCGCG
RBS            M  G  S  S  H  H  H  H  H  H  S  S  G  L  V  P  R  G

GCAGCCATATGGCTAGCATGACTGGTGGACAGCAAATGGGTCGCGGATCCATGGGTAAGGGAGAA
S  H  M  A  S  M  T  G  G  Q  Q  M  G  R  G  S  M  G  K  G  E  E

GAACTTTTCACTGGAGTTGTCCCAATTCTTGTTGAATTAGATGGTGATGTTAATGGGCACAAATT
L  F  T  G  V  V  P  I  L  V  E  L  D  G  D  V  N  G  H  K  F  S

TTCTGTCAGTGGAGAGGGTGAAGGTGATGCAACATACGGAAAACTTACCCTTAAATTTATTTGCA
V  S  G  E  G  E  G  D  A  T  Y  G  K  L  T  L  K  F  I  C  T  T

CTACTGGAAAGCTTCCTGTTCCTTGGCCAACACTTGTCACTACTCTTACTTATGGTGTTCAATGC
G  K  L  P  V  P  W  P  T  L  V  T  T  L  T  Y  G  V  Q  C  F  S

TTTTCAAGATACCCAGATCATATGAAGCGGCACGACTTCTTCAAGAGCGCCATGCCTGAGGGATA
R  Y  P  D  H  M  K  R  H  D  F  F  K  S  A  M  P  E  G  Y  V  Q

CGTGCAGGAGAGGACCATCTTCTTCAAGGACGACGGGAACTACAAGACACGTGCTGAAGTCAAGT
E  R  T  I  F  F  K  D  D  G  N  Y  K  T  R  A  E  V  K  F  E  G

TTGAGGGAGACACCCTCGTCAACAGAATCGAGCTTAAGGGAATCGATTTCAAGGAGGACGGAAAC
D  T  L  V  N  R  I  E  L  K  G  I  D  F  K  E  D  G  N  I  L  G

ATCCTCGGCCACAAGTTGGAATACAACTACAACTCCCACAACGTATACATCATGGCAGACAAACA
H  K  L  E  Y  N  Y  N  S  H  N  V  Y  I  M  A  D  K  Q  K  N  G

AAAGAATGGAATCAAAGTTAACTTCAAAATTAGACACAACATTGAAGATGGAAGCGTTCAACTAG
I  K  V  N  F  K  I  R  H  N  I  E  D  G  S  V  Q  L  A  D  H  Y

CAGACCATTATCAACAAAATACTCCAATTGGCGATGGCCCTGTCCTTTTACCAGACAACCATTAC
Q  Q  N  T  P  I  G  D  G  P  V  L  L  P  D  N  H  Y  L  S  T  Q

CTGTCCACACAATCTGCCCTTTCGAAAGATCCCAACGAAAAGAGAGACCACATGGTCCTTCTTGA
S  A  L  S  K  D  P  N  E  K  R  D  H  M  V  L  L  E  F  V  T  A

GTTTGTAACAGCTGCTGGGATTACACATGGCATGGATGAACTATACAAATGACTCGAG
A  G  I  T  H  G  M  D  E  L  Y  K  *                  XhoI
```

(B)

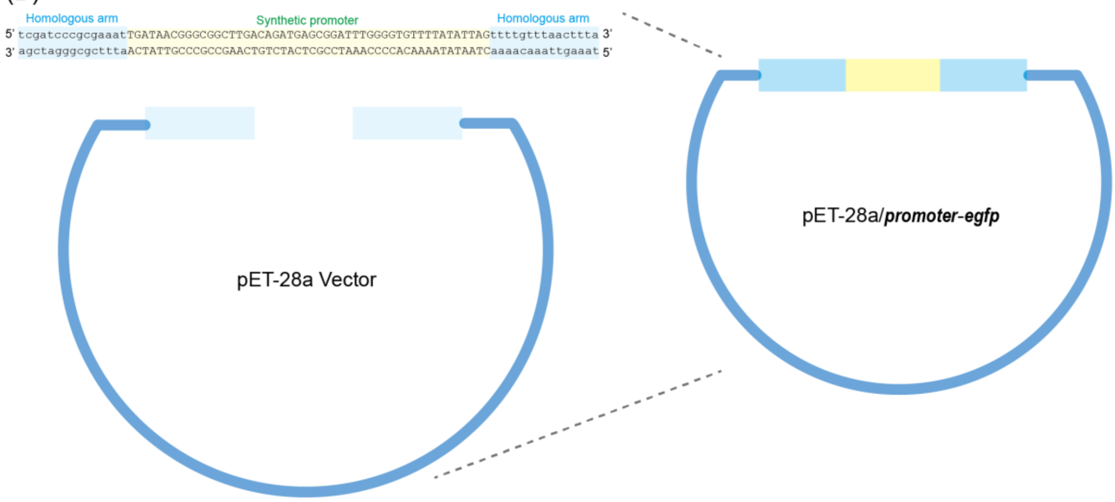

Figure S11 Architecting plasmids carrying different promoters

(A) The expression cassette for *egfp*. (B) Insertion of promoters to pET-28a vector by one-step cloning.

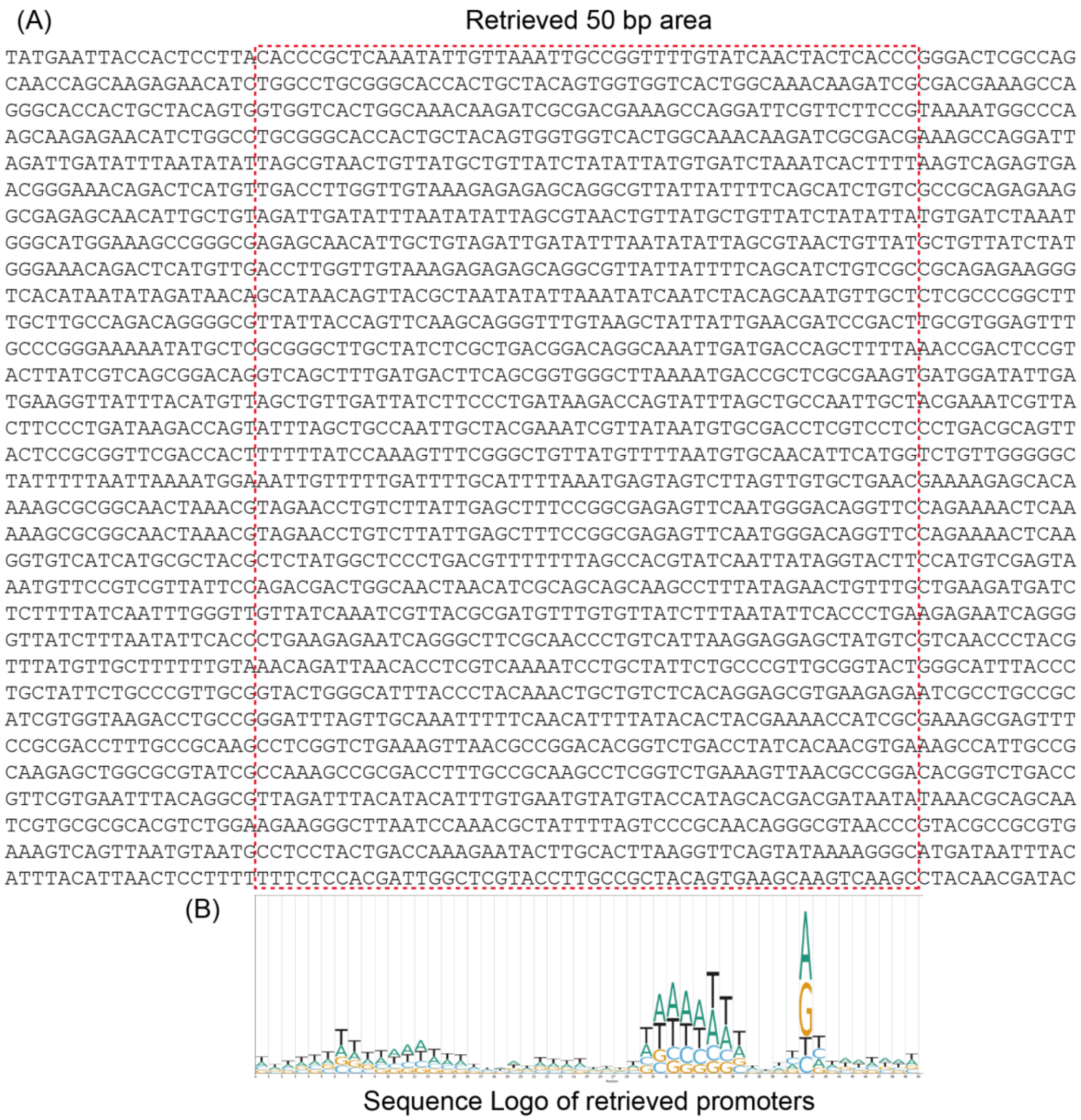

Figure S12 Adjusting promoter length in RegulonDB

(A) Promoters in RegulonDB were retrieved from 18 to 68 according to -35 and -10 motifs' position. (B) Sequence logo of promoters in RegulonDB [5].

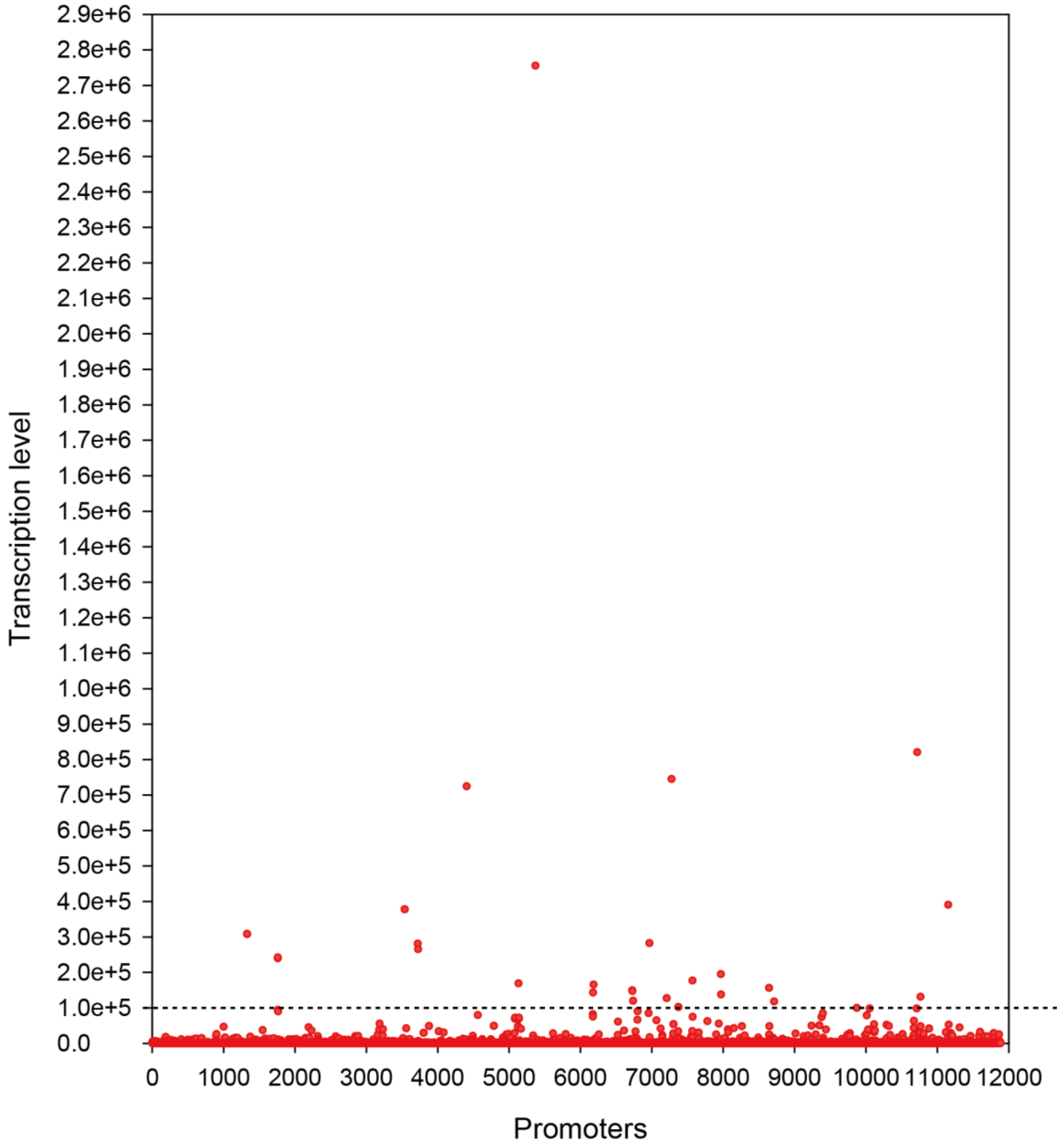

Figure S13 Visualization of promoter transcription level in natural *E. coli* promoter dataset [2]

Supplementary Tables

Table S1 Comparison of iPromoter-CLA[1] with our built PromoR for predicting promoter true or fake.

	SN	SP	ACC	MCC	AUC
iPromoter-CLA (RegulonDB)	0.8687	0.8513	0.8600	0.7211	0.9291
This study (RegulonDB)	0.9343	0.7844	0.8603	0.7279	0.9342
This study (RegulonDB+NDB)	0.8782	0.8938	0.8861	0.7722	0.9527

Table S2 Training result of machine learning models.

**GBR model learned from the promoters with constraint -35 and -10 motifs**				
learning_rate=0.1<br>PCC=0.00977	learning_rate=0.01<br>PCC=0.02219	learning_rate=0.001<br>PCC=0.005636	learning_rate=0.0001<br>PCC=0.002841	learning_rate=0.00001<br>PCC=0.004605
learning_rate=0.01<br>n_estimators=10<br>PCC=0.005331	learning_rate=0.01<br>n_estimators=100<br>PCC=0.0221942	learning_rate=0.01<br>n_estimators=1000<br>PCC=0.00291	learning_rate=0.01<br>n_estimators=10000<br>PCC=0.0013282	learning_rate=0.01<br>n_estimators=100000<br>PCC=0.0013284
learning_rate=0.01<br>n_estimators=10<br>max_depth=1<br>PCC=0.068595	learning_rate=0.01<br>n_estimators=10<br>max_depth=3<br>0.022194	learning_rate=0.01<br>n_estimators=10<br>max_depth=5<br>0.0001494	learning_rate=0.01<br>n_estimators=10<br>max_depth=7<br>0.0004339	learning_rate=0.01<br>n_estimators=10<br>max_depth=9<br>0.001839
learning_rate=0.01<br>n_estimators=10<br>max_depth=1<br>min_samples_leaf=1<br>PCC=0.06859532	learning_rate=0.01<br>n_estimators=10<br>max_depth=1<br>min_samples_leaf=2<br>PCC=0.06859532	learning_rate=0.01<br>n_estimators=10<br>max_depth=1<br>min_samples_leaf=3<br>PCC=0.06859532	learning_rate=0.01<br>n_estimators=10<br>max_depth=1<br>min_samples_leaf=4<br>PCC=0.06859532	learning_rate=0.01<br>n_estimators=10<br>max_depth=1<br>min_samples_leaf=5<br>PCC=0.06859532
**GBR model learned from NDB**				
learning_rate=0.1<br>PCC=0.048108	learning_rate=0.01<br>PCC=0.02227	learning_rate=0.001<br>PCC=0.015044	learning_rate=0.0001<br>PCC=0.013876	learning_rate=0.00001<br>PCC=0.0121441
learning_rate=0.1<br>n_estimators=10<br>PCC=0.0236072	learning_rate=0.1<br>n_estimators=100<br>PCC=0.0480803	learning_rate=0.1<br>n_estimators=1000<br>PCC=0.05822	learning_rate=0.1<br>n_estimators=10000<br>PCC=0.03325	learning_rate=0.1<br>n_estimators=100000<br>PCC=0.0269693
learning_rate=0.1<br>n_estimators=1000<br>max_depth=1<br>PCC=0.0385118	learning_rate=0.1<br>n_estimators=1000<br>max_depth=3<br>PCC=0.05822	learning_rate=0.1<br>n_estimators=1000<br>max_depth=5<br>PCC=0.04358	learning_rate=0.1<br>n_estimators=1000<br>max_depth=7<br>PCC=0.0435597	learning_rate=0.1<br>n_estimators=1000<br>max_depth=9<br>PCC=0.0241127
learning_rate=0.1<br>n_estimators=1000	learning_rate=0.1<br>n_estimators=1000	learning_rate=0.1<br>n_estimators=1000	learning_rate=0.1<br>n_estimators=1000	learning_rate=0.1<br>n_estimators=1000

max_depth=3<br>min_samples_leaf=1<br>PCC=0.058292	max_depth=3<br>min_samples_leaf=2<br>PCC=0.058292	max_depth=3<br>min_samples_leaf=3<br>PCC=0.058292	max_depth=3<br>min_samples_leaf=4<br>PCC=0.058292	max_depth=3<br>min_samples_leaf=5<br>PCC=0.058292
**ENR model learned from the promoters with constraint -35 and -10 motifs**				
alpha=0.1<br>max_iter=1000000<br>l1_ratio=0.5<br>PCC=0.057178	alpha=0.01<br>max_iter=1000000<br>l1_ratio=0.5<br>PCC=0.007166	alpha=0.001<br>max_iter=1000000<br>l1_ratio=0.5<br>PCC=0.0002562	alpha=0.0001<br>max_iter=1000000<br>l1_ratio=0.5<br>PCC=0.0001225	alpha=0.00001<br>max_iter=1000000<br>l1_ratio=0.5<br>PCC=0.0001305
**ENR model learned from NDB**				
alpha=0.1<br>max_iter=1000000<br>l1_ratio=0.5<br>PCC=0	alpha=0.01<br>max_iter=1000000<br>l1_ratio=0.5<br>PCC=0	alpha=0.001<br>max_iter=1000000<br>l1_ratio=0.5<br>PCC=0	alpha=0.0001<br>max_iter=1000000<br>l1_ratio=0.5<br>PCC=0	alpha=0.00001<br>max_iter=1000000<br>l1_ratio=0.5<br>PCC=0

PCC: Pearson correlation coefficient
GBR: gradient boosted trees
ENR: elastic net regression
We used default parameters for training otherwise cleared indicated. The promoters with constraint -35 and -10 motifs were from dataset used to train PromoNet; NDB is a dataset organized by Thomason et al.[2]

Table S3 Primers used in this study.

Primers	Sequences (5′-3′)	Remarks
Pf	ATTTCGCGGGATCGAGATCTCGATCCT	Forward primer for linearizing pET-28a(+)
Pr	TTTTGTTTAACTTTAAGAAGGAGATATACCA	Reverse primer for linearizing pET-28a(+)
R1f	TAAAATTAAACGGGCGTTGTCGCTTTATAAAAACATGTGTTTGCCACAGCTTTTGTTTAACTTTA	Forward primer for promoter R1
R1r	GCTGTGGCAAACACATGTTTTTATAAAGCGACAACGCCCGTTTAATTTTAATTTCGCGGGATCGA	Reverse primer for promoter R1
R2f	GCTGATCTTACTAGTTGTAAGGTTGCCGAACCATGGTAGGTTCTTACGTTTTTTGTTTAACTTTA	Forward primer for promoter R2
R2r	AACGTAAGAACCTACCATGGTTCGGCAACCTTACAACTAGTAAGATCAGCATTTCGCGGGATCGA	Reverse primer for promoter R2
R3f	ACCTGGTGGCAAATGCAAGACACTTGGTGTACATGGTAGTCTGACGCGTCTTTTGTTTAACTTTA	Forward primer for promoter R3
R3r	GACGCGTCAGACTACCATGTACACCAAGTGTCTTGCATTTGCCACCAGGTATTTCGCGGGATCGA	Reverse primer for promoter R3
R4f	GACACTTTGTATTTCTTGAGCTTCTCACTGTTAACCCTATGTTTACCTGTTTTTGTTTAACTTTA	Forward primer for promoter R4
R4r	ACAGGTAAACATAGGGTTAACAGTGAGAAGCTCAAGAAATACAAAGTGTCATTTCGCGGGATCGA	Reverse primer for promoter R4
R5f	CATTAGGCAATCTATTGATGCGAACCGTCAACAAATGAGGTTCCCAACATTTTTGTTTAACTTTA	Forward primer for promoter R5
R5r	ATGTTGGGAACCTCATTTGTTGACGGTTCGCATCAATAGATTGCCTAATGATTTCGCGGGATCGA	Reverse primer for promoter R5
R6f	AGGAAACATATTCCATTGACATTAAGACGCGCAGTGGGTAGACTTAAATCTTTTGTTTAACTTTA	Forward primer for promoter R6
R6r	GATTTAAGTCTACCCACTGCGCGTCTTAATGTCAATGGAATATGTTTCCTATTTCGCGGGATCGA	Reverse primer for promoter R6
R7f	TGATAACGGGCGGCTTGACAGATGAGCGGATTTGGGGTGTTTTATATTAGTTTTGTTTAACTTTA	Forward primer for promoter R7
R7r	CTAATATAAAACACCCCAAATCCGCTCATCTGTCAAGCCGCCCGTTATCAATTTCGCGGGATCGA	Reverse primer for promoter R7
R8f	CCGCGCTTTGGGTCATTGGACACATAGGGTTCCCCAGCGTGACTTGTGTTTTTTGTTTAACTTTA	Forward primer for promoter R8

R8r	AACACAAGTCACGCTGGGGAACCCTATGTGTCCAATGACCCAAAGCGC GGATTTCGCGGGATCGA	Reverse primer for promoter R8
R9f	AATAATCCATCAAGGTTGAGTCATAATTCTCAGTTATTAAAATGGCAGAT TTTTGTTTAACTTTA	Forward primer for promoter R9
R9r	ATCTGCCATTTTAATAACTGAGAATTATGACTCAACCTTGATGGATTATTA TTTCGCGGGATCGA	Reverse primer for promoter R9
R10f	TATTCTACACGGTATGGACCCACACCGTTCCCAGGTACCGTTCCCTAATC TTTTGTTTAACTTTA	Forward primer for promoter R10
R10r	GATTAGGGAACGGTACCTGGGAACGGTGTGGGTCCATACCGTGTAGAAT AATTTCGCGGGATCGA	Reverse primer for promoter R10
R11f	TGCTGATCCTGGCGTTTGGACAGACGGCGATTGTGTGCGGGCGAAGTT GCTTTTGTTTAACTTTA	Forward primer for promoter R11
R11r	GCAACTTCGCCCGCACACAATCGCCGTCTGTCCAAACGCCAGGATCAG CAATTTCGCGGGATCGA	Reverse primer for promoter R11
R12f	CTGGCTCTGGGAAATTGACATTTTTCGACCGTTGTCTGGTTAATGATGG ATTTTGTTTAACTTTA	Forward primer for promoter R12
R12r	TCCATCATTAACCAGACAACGGTCGAAAAATGTCAATTTCCCAGAGCCA GATTTCGCGGGATCGA	Reverse primer for promoter R12
R13f	TTAAATTTTTGCCATATTGCACATAGACAAAAAGATGCTCTTACAGAAAC TTTTGTTTAACTTTA	Forward primer for promoter R13
R13r	GTTTCTGTAAGAGCATCTTTTTGTCTATGTGCAATATGGCAAAAATTTAA ATTTCGCGGGATCGA	Reverse primer for promoter R13
R14f	TTTCCAGCAACACATTGACACTGATAGACGTCCGGCCGCAATTACATATT TTTTGTTTAACTTTA	Forward primer for promoter R14
R14r	AATATGTAATTGCGGCCGGACGTCTATCAGTGTCAATGTGTTGCTGGAA AATTTCGCGGGATCGA	Reverse primer for promoter R14
R15f	AACCAAGTCAAACGTGGACGCAAAAATGATTGGGTTTTATAGTTGACAT TTTTTGTTTAACTTTA	Forward primer for promoter R15
R15r	AATGTCAACTATAAAACCCAATCATTTTTGCGTCCACGTTTGACTTGGTT ATTTCGCGGGATCGA	Reverse primer for promoter R15
R16f	ACTGACGATGCAATATCTTCTCCGGTCATATTAATGAAGCTTTACAAAGA TTTTGTTTAACTTTA	Forward primer for promoter R16
R16r	TCTTTGTAAAGCTTCATTAATATGACCGGAGAAGATATTGCATCGTCAGT ATTTCGCGGGATCGA	Reverse primer for promoter R16
R17f	GGACGCCAGTAAAGGTACATCCCTGAACCCGTTTAATAGTGTGACCAAA	Forward primer for promoter R17

	TTTTTGTTTAACTTTA	
R17r	ATTTGGTCACACTATTAAACGGGTTCAGGGATGTACCTTTACTGGCGTCCATTTCGCGGGATCGA	Reverse primer for promoter R17
R18f	TATGAGCCGGCCCTATTGACGATCGTGTTACAGAGGCCGGTACCCTGGGGTTTTGTTTAACTTTA	Forward primer for promoter R18
R18r	CCCCAGGGTACCGGCCTCTGTAACACGATCGTCAATAGGGCCGGCTCATAATTTCGCGGGATCGA	Reverse primer for promoter R18
R19f	TCTACCCCCGGGTTTTGAACCATCTGCCCAAAACCGGCCAGTCCCGTTAATTTTGTTTAACTTTA	Forward primer for promoter R19
R19r	TTAACGGGACTGGCCGGTTTTGGGCAGATGGTTCAAAACCCGGGGGTAGAATTTCGCGGGATCGA	Reverse primer for promoter R19
R20f	GCCTGTTGAAAGAGTTGACGTTGACGGCACCTGAAAACAGATTACAGTGTTTTTGTTTAACTTTA	Forward primer for promoter R20
R20r	ACACTGTAATCTGTTTTCAGGTGCCGTCAACGTCAACTCTTTCAACAGGCATTTCGCGGGATCGA	Reverse primer for promoter R20
R21f	GCGCGTCCACCTCATTACCCCCCCTTGAGGCAAATTCAGGGTATTGTTTTTTTTGTTTAACTTTA	Forward primer for promoter R21
R21r	AAAACAATACCCTGAATTTGCCTCAAGGGGGGGTAATGAGGTGGACGCGCATTTCGCGGGATCGA	Reverse primer for promoter R21
R22f	TGGCCATTATGCCACTACAGCAAGAGGCGTCTGTACGAGCTATAATTTGTTTTTGTTTAACTTTA	Forward primer for promoter R22
R22r	ACAAATTATAGCTCGTACAGACGCCTCTTGCTGTAGTGGCATAATGGCCAATTTCGCGGGATCGA	Reverse primer for promoter R22
R23f	AGGGTCCAACAACGGTTGTAGGCCAATTTATACTGAGTCCTTCAATATTTTTTTGTTTAACTTTA	Forward primer for promoter R23
R23r	AAATATTGAAGGACTCAGTATAAATTGGCCTACAACCGTTGTTGGACCCTATTTCGCGGGATCGA	Reverse primer for promoter R23
R24f	AGCTGTAATGTAAATTTCCTATCGCACAAAAGCGGTGCATATTACGCGCCTTTTGTTTAACTTTA	Forward primer for promoter R24
R24r	GGCGCGTAATATGCACCGCTTTTGTGCGATAGGAAATTTACATTACAGCTATTTCGCGGGATCGA	Reverse primer for promoter R24
R25f	TCAGCGTCGCCCGTAAGTAAGGGACCCGCCATAATGTTATAATATAGTGTTTTTGTTTAACTTTA	Forward primer for promoter R25
R25r	ACACTATATTATAACATTATGGCGGGTCCCTTACTTACGGGCGACGCTGAATTTCGCGGGATCGA	Reverse primer for promoter R25

R26f	ACGCAATCATGATAGCATTGAATTGACCTAATTCGACAAAAATAGTAATTTTTTGTTTAACTTTA	Forward primer for promoter R26
R26r	AATTACTATTTTTGTCGAATTAGGTCAATTCAATGCTATCATGATTGCGTATTTCGCGGGATCGA	Reverse primer for promoter R26
R27f	CGGATGGTTTGCACCCCCCGCAAAGACCCAATGTGAAGTATACTGATAGTTTTTGTTTAACTTTA	Forward primer for promoter R27
R27r	ACTATCAGTATACTTCACATTGGGTCTTTGCGGGGGGTGCAAACCATCCGATTTCGCGGGATCGA	Reverse primer for promoter R27
R28f	TTGTAAAAATTTACTTCGGTAATTGTTAAGGGGCGGAACTATTATGCTCCTTTTGTTTAACTTTA	Forward primer for promoter R28
R28r	GGAGCATAATAGTTCCGCCCCTTAACAATTACCGAAGTAAATTTTTACAAATTTCGCGGGATCGA	Reverse primer for promoter R28
R29f	CGAACGCTCAAAATATTGTGGTTTTCAACAACAGATGCCAATCGCAGCCTTTTTGTTTAACTTTA	Forward primer for promoter R29
R29r	AGGCTGCGATTGGCATCTGTTGTTGAAAACCACAATATTTTGAGCGTTCGATTTCGCGGGATCGA	Reverse primer for promoter R29
R30f	GTACCCATTGTTAGAAATAAAGAAGGCTGTTAATCGCCAAAATCAAAATTTTTTGTTTAACTTTA	Forward primer for promoter R30
R30r	AATTTTGATTTTGGCGATTAACAGCCTTCTTTATTTCTAACAATGGGTACATTTCGCGGGATCGA	Reverse primer for promoter R30
R31f	CCCCAGATAGCCACTTCCCTTTACTGCGGCCTGTTCTGTTTAATAGATGTTTTTGTTTAACTTTA	Forward primer for promoter R31
R31r	ACATCTATTAAACAGAACAGGCCGCAGTAAAGGGAAGTGGCTATCTGGGGATTTCGCGGGATCGA	Reverse primer for promoter R31
R32f	AGACAGAATGACCCCTGTAAAATTTGTGAAGTGGACCCAATACAGTAACATTTTGTTTAACTTTA	Forward primer for promoter R32
R32r	TGTTACTGTATTGGGTCCACTTCACAAATTTTACAGGGGTCATTCTGTCTATTTCGCGGGATCGA	Reverse primer for promoter R32
R33f	AGTAAAGACCTGTTTGATCTCCTGCCGCAAAATTTGAGGTGTTTACACCCTTTTGTTTAACTTTA	Forward primer for promoter R33
R33r	GGGTGTAAACACCTCAAATTTTGCGGCAGGAGATCAAACAGGTCTTTACTATTTCGCGGGATCGA	Reverse primer for promoter R33
R34f	TGGCCCGGCCAGTAAACGCCCTGACCCCGTCAATTGCTAAATCTAGCCGTTTTTGTTTAACTTTA	Forward primer for promoter R34
R34r	ACGGCTAGATTTAGCAATTGACGGGGTCAGGGCGTTTACTGGCCGGGCC	Reverse primer for promoter R34

	AATTTCGCGGGATCGA	
R35f	GCGAAACGACGCCTCACACATTGATCCGCACCAAGCGCTCTTAGAAAATGTTTTGTTTAACTTTA	Forward primer for promoter R35
R35r	CATTTTCTAAGAGCGCTTGGTGCGGATCAATGTGTGAGGCGTCGTTTCGCATTTCGCGGGATCGA	Reverse primer for promoter R35
R36f	TGATGTTTCTTTGGTTGAGTTGCAGCCCGACTGCAGACACTTACATAACATTTTGTTTAACTTTA	Forward primer for promoter R36
R36r	TGTTATGTAAGTGTCTGCAGTCGGGCTGCAACTCAACCAAAGAAACATCAATTTCGCGGGATCGA	Reverse primer for promoter R36
R37f	AAGCACGTCAGAGGTAGGCCCTGAAGCCCGCAACCCCCAATTGTGCTAGTTTTTGTTTAACTTTA	Forward primer for promoter R37
R37r	ACTAGCACAATTGGGGGTTGCGGGCTTCAGGGCCTACCTCTGACGTGCTTATTTCGCGGGATCGA	Reverse primer for promoter R37
R38f	TTACGGTCTTAAGCATGGCTTTGAATGACCTGACGTTTAATTTGTAGTTTTTTTGTTTAACTTTA	Forward primer for promoter R38
R38r	AAACTACAAATTAAACGTCAGGTCATTCAAAGCCATGCTTAAGACCGTAAATTTCGCGGGATCGA	Reverse primer for promoter R38
R39f	AGAGCTAAATCCATTTAGCGATGGTCAAGAGTCAGGTCCCCTGCATTTTATTTTGTTTAACTTTA	Forward primer for promoter R39
R39r	TAAAATGCAGGGGACCTGACTCTTGACCATCGCTAAATGGATTTAGCTCTATTTCGCGGGATCGA	Reverse primer for promoter R39
R40f	TGTGGTTGACCCCACAACAGGCACAGAGCAATAAATGGGTTATAGATGGTTTTTGTTTAACTTTA	Forward primer for promoter R40
R40r	ACCATCTATAACCCATTTATTGCTCTGTGCCTGTTGTGGGGTCAACCACAATTTCGCGGGATCGA	Reverse primer for promoter R40
R41f	TAACGACTTATTTGGTTGGAAATTCCTGTCCAAGGGTGGTGTGAAACCCCTTTTGTTTAACTTTA	Forward primer for promoter R41
R41r	GGGGTTTCACACCACCCTTGGACAGGAATTTCCAACCAAATAAGTCGTTAATTTCGCGGGATCGA	Reverse primer for promoter R41
R42f	TGTTCCTAATGAGTATCGTTCAACATGCTTTTCATGTTATATTAGGCGCCTTTTGTTTAACTTTA	Forward primer for promoter R42
R42r	GGCGCCTAATATAACATGAAAAGCATGTTGAACGATACTCATTAGGAACAATTTCGCGGGATCGA	Reverse primer for promoter R42
R43f	TCCGTTATTTAATCTGGCCAACATTTCGTAAGTGCTCGAGTAAAATCCAATTTTGTTTAACTTTA	Forward primer for promoter R43

R43r	TTGGATTTTACTCGAGCACTTACGAAATGTTGGCCAGATTAAATAACGGAATTTCGCGGGATCGA	Reverse primer for promoter R43
R44f	TTTCAGCTAATGGGTTGCACTTATAAGCCCCCTATACCCTAACCGTAGCTTTTTGTTTAACTTTA	Forward primer for promoter R44
R44r	AGCTACGGTTAGGGTATAGGGGGCTTATAAGTGCAACCCATTAGCTGAAAATTTCGCGGGATCGA	Reverse primer for promoter R44
R45f	TGATCCATAACCATCTTGAGTTTTACCGCTACCCCATCAATGGTTACACATTTTGTTTAACTTTA	Forward primer for promoter R45
R45r	TGTGTAACCATTGATGGGGTAGCGGTAAAACTCAAGATGGTTATGGATCAATTTCGCGGGATCGA	Reverse primer for promoter R45
R46f	CATCTGCGGGACTTGAAAAGGCCATTAACGTGTGCTGTATTGTTGTATCATTTTGTTTAACTTTA	Forward primer for promoter R46
R46r	TGATACAACAATACAGCACACGTTAATGGCCTTTTCAAGTCCCGCAGATGATTTCGCGGGATCGA	Reverse primer for promoter R46
R47f	CCTGAAATAAATACCCATAAGCCTTTACAATTTTTGTGCAATCTTCATCTTTTTGTTTAACTTTA	Forward primer for promoter R47
R47r	AGATGAAGATTGCACAAAAATTGTAAAGGCTTATGGGTATTTATTTCAGGATTTCGCGGGATCGA	Reverse primer for promoter R47
R48f	CGGACCCAACTTCCTTGGACATATGTGTCGCTCCTGGCCAAAGCAACTTCTTTTGTTTAACTTTA	Forward primer for promoter R48
R48r	GAAGTTGCTTTGGCCAGGAGCGACACATATGTCCAAGGAAGTTGGGTCCGATTTCGCGGGATCGA	Reverse primer for promoter R48
R49f	TCTTTTTATGAACATTCTTAGTCTCGGTCCATACTTGCTCCTCACAATGCTTTTGTTTAACTTTA	Forward primer for promoter R49
R49r	GCATTGTGAGGAGCAAGTATGGACCGAGACTAAGAATGTTCATAAAAAGAATTTCGCGGGATCGA	Reverse primer for promoter R49
R50f	CGATGTCAACTTGATAAGGCCCACAGCCCTGATTTGCTATATTGACAGATTTTTGTTTAACTTTA	Forward primer for promoter R50
R50r	ATCTGTCAATATAGCAAATCAGGGCTGTGGGCCTTATCAAGTTGACATCGATTTCGCGGGATCGA	Reverse primer for promoter R50
P1f	CACGCGGCCGCCACCTTGACAAAGTACAATAATTGATTATAATAGTCTAGTTTTGTTTAACTTTA	Forward primer for promoter P1
P1r	CTAGACTATTATAATCAATTATTGTACTTTGTCAAGGTGGCGGCCGCGTGATTTCGCGGGATCGA	Reverse primer for promoter P1
P2f	ATGCAAAACGTCTAGTTGACAAAAGCGATACACTTGGTTATAATCATGC	Forward primer for promoter P2

	GTTTTGTTTAACTTTA	
P2r	CGCATGATTATAACCAAGTGTATCGCTTTTGTCAACTAGACGTTTTGCAT ATTTCGCGGGATCGA	Reverse primer for promoter P2
P3f	CTCCACTTGTCCTCATTGACAAAATCCGCATATCTGCGTATAATTCTCTTT TTTGTTTAACTTTA	Forward primer for promoter P3
P3r	AAGAGAATTATACGCAGATATGCGGATTTTGTCAATGAGGACAAGTGGA GATTTCGCGGGATCGA	Reverse primer for promoter P3
P4f	ATTATGTTTAGAACCTTGACATTTTCCCAGTGTATGCATATAATAGGTTAT TTTGTTTAACTTTA	Forward primer for promoter P4
P4r	TAACCTATTATATGCATACACTGGGAAAATGTCAAGGTTCTAAACATAAT ATTTCGCGGGATCGA	Reverse primer for promoter P4
P5f	CAAGTGAGAGTATAATTGACAATAAAATTGCCATCGGTATAATAGTATAG TTTTGTTTAACTTTA	Forward primer for promoter P5
P5r	CTATACTATTATACCGATGGCAATTTTATTGTCAATTATACTCTCACTTGAT TTCGCGGGATCGA	Reverse primer for promoter P5
P6f	TCGAGCAGTCGGGGCTTGACAAGACAAGTGCGGGACCTATAATAGTATA GTTTTGTTTAACTTTA	Forward primer for promoter P6
P6r	CTATACTATTATAGGTCCCGCACTTGTCTTGTCAAGCCCCGACTGCTCGA ATTTCGCGGGATCGA	Reverse primer for promoter P6
P7f	AGCCTTATGGAAATTTGACAAGGTGAGTGGCCTAACGCTATAATAGGTG TTTTTGTTTAACTTTA	Forward primer for promoter P7
P7r	ACACCTATTATAGCGTTAGGCCACTCACCTTGTCAAATTTCCATAAGGCT ATTTCGCGGGATCGA	Reverse primer for promoter P7
P8f	CTAGAAATCGTTAGATTGACAAAAGATGTCCTTAGCCATATAATAGGTCG TTTTGTTTAACTTTA	Forward primer for promoter P8
P8r	CGACCTATTATATGGCTAAGGACATCTTTTGTCAATCTAACGATTTCTAGA TTTCGCGGGATCGA	Reverse primer for promoter P8
P9f	AATTGTTTTAAAACCTTGACAATTGTTATGCGTGGACGTATAATAATTTTT TTTGTTTAACTTTA	Forward primer for promoter P9
P9r	AAAATTATTATACGTCCACGCATAACAATTGTCAAGGTTTTAAAACAATT ATTTCGCGGGATCGA	Reverse primer for promoter P9
P10f	GTAATCAGTCGGCTCTTGACAATATTCTAAGTGAACTTTATAATTTATACT TTTGTTTAACTTTA	Forward primer for promoter P10
P10r	GTATAAATTATAAAGTTCACTTAGAATATTGTCAAGAGCCGACTGATTAC ATTTCGCGGGATCGA	Reverse primer for promoter P10

P11f	TAGTCACATGCGTGCTTGACAAAAGGGTATCGGTGGTTATAATATATCCG TTTTGTTTAACTTTA	Forward primer for promoter P11
P11r	CGGATATATTATAACCACCGATACCCTTTTGTCAAGCACGCATGTGACTA ATTTCGCGGGATCGA	Reverse primer for promoter P11
P12f	TGTCAACTATAAGGCTTGACAATAAGTGTAAAAGACCTTATAATAGGTGT TTTTGTTTAACTTTA	Forward primer for promoter P12
P12r	ACACCTATTATAAGGTCTTTTACACTTATTGTCAAGCCTTATAGTTGACA ATTTCGCGGGATCGA	Reverse primer for promoter P12
P13f	TATGACTATGAAACTTTGACAAAGTGTGTTAAGTGGCTATAATACGCTAG TTTTGTTTAACTTTA	Forward primer for promoter P13
P13r	CTAGCGTATTATAGCCACTTAACACACTTTGTCAAAGTTTCATAGTCATA ATTTCGCGGGATCGA	Reverse primer for promoter P13
P14f	GGCGTGGCGCAGATATTGACACTAACATACCCCCGGGTATAATAGCACG GTTTTGTTTAACTTTA	Forward primer for promoter P14
P14r	CCGTGCTATTATACCCGGGGGTATGTTAGTGTCAATATCTGCGCCACGCC ATTTCGCGGGATCGA	Reverse primer for promoter P14
P15f	TCCCCCCAAACCGATTGACAAGGTGCGGGTAATTCAAGTATAATTACCT TTTTTGTTTAACTTTA	Forward primer for promoter P15
P15r	AAGGTAATTATACTTGAATTACCCGCACCTTGTCAATCGGTTTGGGGGG AATTTCGCGGGATCGA	Reverse primer for promoter P15
P16f	TGTACGGTTGCAGGCTTGACAAAAGACGCTACCTGCTTATAATGCACTA ATTTTGTTTAACTTTA	Forward primer for promoter P16
P16r	TTAGTGCATTATAAGCAGGTAGCGTCTTTTGTCAAGCCTGCAACCGTAC AATTTCGCGGGATCGA	Reverse primer for promoter P16
P17f	AATTCTTATGTCATGTTGACAGTTTGTATGTGTACTGGTATAATCCGGGCT TTTGTTTAACTTTA	Forward primer for promoter P17
P17r	GCCCGGATTATACCAGTACACATACAAACTGTCAACATGACATAAGAAT TATTTCGCGGGATCGA	Reverse primer for promoter P17
P18f	AAAAGGGAAGACATGTTGACAATAAACGAATGATTTCTATAATAGCACG ATTTTGTTTAACTTTA	Forward primer for promoter P18
P18r	TCGTGCTATTATAGAAATCATTCGTTTATTGTCAACATGTCTTCCCTTTTA TTTCGCGGGATCGA	Reverse primer for promoter P18
P19f	GATAAAGCGCGACAATTGACAAAAGTCGCGCACGCGGTATAATACATCG TTTTTGTTTAACTTTA	Forward primer for promoter P19
P19r	ACGATGTATTATACCGCGTGCGCGACTTTTGTCAATTGTCGCGCTTTATC	Reverse primer for promoter P19

	ATTTCGCGGGATCGA	
P20f	AATGAACCCCTTACCTTGACAAAATTCAATTGGGCCTTATAATGGCCCTT TTTTGTTTAACTTTA	Forward primer for promoter P20
P20r	AAGGGCCATTATAAGGCCCAATTGAATTTTGTCAAGGTAAGGGGTTCAT TATTTCGCGGGATCGA	Reverse primer for promoter P20
P21f	AAGAGCGCTATCTGCTTGACAATTGATGTGGAGCGGGGTATAATAGGCG TTTTTGTTTAACTTTA	Forward primer for promoter P21
P21r	ACGCCTATTATACCCCGCTCCACATCAATTGTCAAGCAGATAGCGCTCTT ATTTCGCGGGATCGA	Reverse primer for promoter P21
P22f	GAGGGCATCTGCGGCTTGACAAAAGTCTACTTAGAACTATAATTGAGTT TTTTTGTTTAACTTTA	Forward primer for promoter P22
P22r	AAACTCAATTATAGTTCTAAGTAGACTTTTGTCAAGCCGCAGATGCCCTC ATTTCGCGGGATCGA	Reverse primer for promoter P22
P23f	GTTGACTTAAGATGATTGACAAGAGATCTAGTGATAATATAATATGAATT TTTTGTTTAACTTTA	Forward primer for promoter P23
P23r	AATTCATATTATATTATCACTAGATCTCTTGTCAATCATCTTAAGTCAACAT TTCGCGGGATCGA	Reverse primer for promoter P23
P24f	GCTTGTTAAAGTGTGTTGACAGAAGATGATGCTATGATATAATATACTGT TTTTGTTTAACTTTA	Forward primer for promoter P24
P24r	ACAGTATATTATATCATAGCATCATCTTCTGTCAACACACTTTAACAAGC ATTTCGCGGGATCGA	Reverse primer for promoter P24
P25f	ACGATTTATTGTTCATTGACAAGAGGGAGACGGGATCTATAATATACTAG TTTTGTTTAACTTTA	Forward primer for promoter P25
P25r	CTAGTATATTATAGATCCCGTCTCCCTCTTGTCAATGAACAATAAATCGTA TTTCGCGGGATCGA	Reverse primer for promoter P25
P26f	TATGAGCAACTGCTCTTGACAAAATAGTTTGACTTGGTATAATCAGCATA TTTTGTTTAACTTTA	Forward primer for promoter P26
P26r	TATGCTGATTATACCAAGTCAAACTATTTTGTCAAGAGCAGTTGCTCATA ATTTCGCGGGATCGA	Reverse primer for promoter P26
P27f	GTGACCGCTCGATTATTGACACAAAGAGTATCCTTAATATAATAGCCTAG TTTTGTTTAACTTTA	Forward primer for promoter P27
P27r	CTAGGCTATTATATTAAGGATACTCTTTGTGTCAATAATCGAGCGGTCAC ATTTCGCGGGATCGA	Reverse primer for promoter P27
P28f	TATGTCGTTCGTCCCTTGACAGAATGACTTAGGTGACTATAATATCTTACT TTTGTTTAACTTTA	Forward primer for promoter P28

P28r	GTAAGATATTATAGTCACCTAAGTCATTCTGTCAAGGGACGAACGACATA ATTTCGCGGGATCGA	Reverse primer for promoter P28
P29f	TTGTGCGTGGGCTGGTTGACAAAAGGGGCTCTGTACTGTATAATTTACC TTTTTGTTTAACTTTA	Forward primer for promoter P29
P29r	AGGTAAATTATACAGTACAGAGCCCCTTTTGTCAACCAGCCCACGCACA AATTTCGCGGGATCGA	Reverse primer for promoter P29
P30f	TTTGTACCCGAACCTTGACATCTAATGGTTACTGAGTGTATAATAGGTGT TTTTGTTTAACTTTA	Forward primer for promoter P30
P30r	ACACCTATTATACACTCAGTAACCATTAGATGTCAAGGTTCGGGTACAA AATTTCGCGGGATCGA	Reverse primer for promoter P30
P31f	TTATTAGCCCTCTCCTTGACAAAAGCTAATGTTTGATTATAATAAGCAGG TTTTGTTTAACTTTA	Forward primer for promoter P31
P31r	CCTGCTTATTATAATCAAACATTAGCTTTTGTCAAGGAGAGGGCTAATAA ATTTCGCGGGATCGA	Reverse primer for promoter P31
P32f	GGGAAACAACTCAGATTGACAAAAGAAGAATACTACGTATAATAGTATT ATTTTGTTTAACTTTA	Forward primer for promoter P32
P32r	TAATACTATTATACGTAGTATTCTTCTTTTGTCAATCTGAGTTGTTTCCCAT TTCGCGGGATCGA	Reverse primer for promoter P32
P33f	AGGGGAACCATGTCGTTGACAAAATGGTCATTCTGTGTATAATACTAACT TTTTGTTTAACTTTA	Forward primer for promoter P33
P33r	AGTTAGTATTATACACAGAATGACCATTTTGTCAACGACATGGTTCCCCT ATTTCGCGGGATCGA	Reverse primer for promoter P33
P34f	AATGATCGAACAGATTTGACAATAATCGTGATAAATGATATAATAGGCTG TTTTGTTTAACTTTA	Forward primer for promoter P34
P34r	CAGCCTATTATATCATTTATCACGATTATTGTCAAATCTGTTCGATCATTAT TTCGCGGGATCGA	Reverse primer for promoter P34
P35f	AATGAAGGTAGTAATTTGACAAAAGCCCTCAGCGCCCGTATAATCATAG TTTTTGTTTAACTTTA	Forward primer for promoter P35
P35r	ACTATGATTATACGGGCGCTGAGGGCTTTTGTCAAATTACTACCTTCATT ATTTCGCGGGATCGA	Reverse primer for promoter P35
P36f	AAGTCTTCGTCATAATTGACATAACCTTCATAAGGGTATATAATCCTCACT TTTGTTTAACTTTA	Forward primer for promoter P36
P36r	GTGAGGATTATATACCCTTATGAAGGTTATGTCAATTATGACGAAGACTT ATTTCGCGGGATCGA	Reverse primer for promoter P36
P37f	CAAATGAAAAAGTGATTGACAATAACCCTAAAGGTGTTATAATATAATCG	Forward primer for promoter P37

	TTTTGTTTAACTTTA	
P37r	CGATTATATTATAACACCTTTAGGGTTATTGTCAATCACTTTTTCATTTGATTTCGCGGGATCGA	Reverse primer for promoter P37
P38f	AGATTCCTACAACCCTTGACAGTTTCGATATCCGTGATATAATACTACCCTTTTGTTTAACTTTA	Forward primer for promoter P38
P38r	GGGTAGTATTATATCACGGATATCGAAACTGTCAAGGGTTGTAGGAATCTATTTCGCGGGATCGA	Reverse primer for promoter P38
P39f	TAGGCACCTGACGAATTGACAGTTGACGGGAACTTCGCTATAATGACTCTTTTTGTTTAACTTTA	Forward primer for promoter P39
P39r	AGAGTCATTATAGCGAAGTTCCCGTCAACTGTCAATTCGTCAGGTGCCTAATTTCGCGGGATCGA	Reverse primer for promoter P39
P40f	CCTTACAACGAAACTTGACAAGGTTCTAGTTACTCACTTATAATACATCGTTTTGTTTAACTTTA	Forward primer for promoter P40
P40r	CGATGTATTATAAGTGAGTAACTAGAACCTTGTCAAGTTTCGTTGTAAGGATTTCGCGGGATCGA	Reverse primer for promoter P40
P41f	AAATTACAAATGCATTTGACAGAACCTCTTTGCACTTTATAATAGTACGATTTTGTTTAACTTTA	Forward primer for promoter P41
P41r	TCGTACTATTATAAAGTGCAAAGAGGTTCTGTCAAATGCATTTGTAATTTATTTCGCGGGATCGA	Reverse primer for promoter P41
P42f	TCTGAAATTTGGGACTTGACATGATGCTAGTTTAAGTGTATAATAGGTGTTTTTGTTTAACTTTA	Forward primer for promoter P42
P42r	ACACCTATTATACACTTAAACTAGCATCATGTCAAGTCCCAAATTTCAGAATTTCGCGGGATCGA	Reverse primer for promoter P42
P43f	TTTGTCTTTAGCAGTTGACATTAGAGGCAACCTAAAGCTATAATAGGTGTTTTTGTTTAACTTTA	Forward primer for promoter P43
P43r	ACACCTATTATAGCTTTAGGTTGCCTCTAATGTCAACTGCTAAAGACAAAATTTCGCGGGATCGA	Reverse primer for promoter P43
P44f	CCTATTGACATTGATTGACATCGTATACAATTGGGACATATAATAGGATATTTTGTTTAACTTTA	Forward primer for promoter P44
P44r	TATCCTATTATATGTCCCAATTGTATACGATGTCAATCAATGTCAATAGGATTTCGCGGGATCGA	Reverse primer for promoter P44
P45f	AGACATTCAGAGTCGTTGACAAAAGCTTATACGACAGATATAATAGGCGTTTTTGTTTAACTTTA	Forward primer for promoter P45
P45r	ACGCCTATTATATCTGTCGTATAAGCTTTTGTCAACGACTCTGAATGTCTATTTCGCGGGATCGA	Reverse primer for promoter P45

P46f	ATAGAAAGCTTTTGTTGACAAAGGTAAGTTTAGGGACGTATAATACAATCTTTTGTTTAACTTTA	Forward primer for promoter P46
P46r	GATTGTATTATACGTCCCTAAACTTACCTTTGTCAACAAAAGCTTTCTATATTTCGCGGGATCGA	Reverse primer for promoter P46
P47f	GCCTCTAGTCGACGATTGACAAAAAGTCAGAAATATGTATAATAGCACTCTTTTGTTTAACTTTA	Forward primer for promoter P47
P47r	GAGTGCTATTATACATATTTCTGACTTTTTGTCAATCGTCGACTAGAGGCATTTCGCGGGATCGA	Reverse primer for promoter P47
P48f	TAAATCGTACCATTTTGACATGGTTACGTAAACTACTCTATAATTCTGTTTTTTGTTTAACTTTA	Forward primer for promoter P48
P48r	AACAGAATTATAGAGTAGTTTACGTAACCATGTCAAAATGGTACGATTTAATTTCGCGGGATCGA	Reverse primer for promoter P48
P49f	CGGCGCTGTGACTGCTTGACAAAGTCCCATCAACCCGGTATAATAGGTGTTTTTGTTTAACTTTA	Forward primer for promoter P49
P49r	ACACCTATTATACCGGGTTGATGGGACTTTGTCAAGCAGTCACAGCGCCGATTTCGCGGGATCGA	Reverse primer for promoter P49
P50f	GCCCCCCCTCCACGATTGACAAAACATCTTCACGTATATATAATATGCGTTTTTGTTTAACTTTA	Forward primer for promoter P50
P50r	ACGCATATTATATATACGTGAAGATGTTTTGTCAATCGTGGAGGGGGGGCATTTCGCGGGATCGA	Reverse primer for promoter P50
P51f	AATGAGTTTGACTGCTTGACAGAAATATAATTTGGCTTTATAATTCTATCTTTTGTTTAACTTTA	Forward primer for promoter P51
P51r	GATAGAATTATAAAGCCAAATTATATTTCTGTCAAGCAGTCAAACTCATTATTTCGCGGGATCGA	Reverse primer for promoter P51
P52f	TGTGAGACCGACAGCTTGACAAAACATATAAACCGAATATAATGCCCGCCTTTTGTTTAACTTTA	Forward primer for promoter P52
P52r	GGCGGGCATTATATTCGGTTTATATGTTTTGTCAAGCTGTCGGTCTCACAATTTCGCGGGATCGA	Reverse primer for promoter P52
P53f	TCGCCGATGGCTATTTTGACAAAAGTCAGACTTATGCTTATAATAGCCTCTTTTGTTTAACTTTA	Forward primer for promoter P53
P53r	GAGGCTATTATAAGCATAAGTCTGACTTTTGTCAAAATAGCCATCGGCGAATTTCGCGGGATCGA	Reverse primer for promoter P53
P54f	GCCCCCTTTGTCGATTGACAAGTTTCTTCAAAAGGACCTATAATGAGGACTTTTGTTTAACTTTA	Forward primer for promoter P54
P54r	GTCCTCATTATAGGTCCTTTTGAAGAAACTTGTCAATCGACAAAGGGGG	Reverse primer for promoter P54

	CATTTCGCGGGATCGA	
P55f	GTGTACTGATGCCCTTGACAAAAAGAGTAACAATGCACTATAATGCACC CTTTTGTTTAACTTTA	Forward primer for promoter P55
P55r	GGGTGCATTATAGTGCATTGTTACTCTTTTTGTCAAGGGCATCAGTACAC ATTTCGCGGGATCGA	Reverse primer for promoter P55
P56f	AAATCTCTACTATCTTGACACAGTTAGCCGACTGAGTTTATAATATGCGT TTTTGTTTAACTTTA	Forward primer for promoter P56
P56r	ACGCATATTATAAACTCAGTCGGCTAACTGTGTCAAGATAGTAGAGATTT ATTTCGCGGGATCGA	Reverse primer for promoter P56
P57f	AAATAGATAAACTCTTGACACAGATCTGGTATTAAGATTATAATGCCGCC TTTTGTTTAACTTTA	Forward primer for promoter P57
P57r	GGCGGCATTATAATCTTAATACCAGATCTGTGTCAAGAGTTTATCTATTTA TTTCGCGGGATCGA	Reverse primer for promoter P57
P58f	GCTTGTTGATCACCTTTGACAAAAGGACTTTCCTTAACTATAATACCGTG TTTTGTTTAACTTTA	Forward primer for promoter P58
P58r	CACGGTATTATAGTTAAGGAAAGTCCTTTTGTCAAAGGTGATCAACAAG CATTTCGCGGGATCGA	Reverse primer for promoter P58
P59f	CATCGACCCTATCATTTGACAAGAGCCCTTCGGTAGCTATAATAGACTGA TTTTGTTTAACTTTA	Forward primer for promoter P59
P59r	TCAGTCTATTATAGCTACCGAAGGGCTCTTGTCAAATGATAGGGTCGATG ATTTCGCGGGATCGA	Reverse primer for promoter P59
P60f	CGAGTTCTACCTTTCTTGACACTAATAAAACTAATGACTATAATCACCGT TTTTGTTTAACTTTA	Forward primer for promoter P60
P60r	ACGGTGATTATAGTCATTAGTTTTATTAGTGTCAAGAAAGGTAGAACTCG ATTTCGCGGGATCGA	Reverse primer for promoter P60
P61f	TTGACGCAATGTAACTTGACAAAAGTGAAGATTGATCCTATAATGAAGC ATTTTGTTTAACTTTA	Forward primer for promoter P61
P61r	TGCTTCATTATAGGATCAATCTTCACTTTTGTCAAGTTACATTGCGTCAA ATTTCGCGGGATCGA	Reverse primer for promoter P61
P62f	CTTAACTCTCATGATTGACAAGGTTTTGCGTCTGGTGTTATAATCCACGT TTTTGTTTAACTTTA	Forward primer for promoter P62
P62r	ACGTGGATTATAACACCAGACGCAAAACCTTGTCAATCATGAGAGTTAA GATTTCGCGGGATCGA	Reverse primer for promoter P62
P63f	TGTGAGAGATAGTGCTTGACAGAATACTTTAACTTAGTTATAATCACCTT TTTTGTTTAACTTTA	Forward primer for promoter P63

P63r	AAGGTGATTATAACTAAGTTAAAGTATTCTGTCAAGCACTATCTCTCACA ATTTCGCGGGATCGA	Reverse primer for promoter P63
P64f	CTCAGGTGGTAGTAGTTGACAAAAGGAGTATACTAGAGTATAATGACCG TTTTTGTTTAACTTTA	Forward primer for promoter P64
P64r	ACGGTCATTATACTCTAGTATACTCCTTTTGTCAACTACTACCACCTGAG ATTTCGCGGGATCGA	Reverse primer for promoter P64
P65f	ATTACAGAGTTTTGCTTGACAAAATGCGATCTGCAGATTATAATAGCGCC TTTTGTTTAACTTTA	Forward primer for promoter P65
P65r	GGCGCTATTATAATCTGCAGATCGCATTTTGTCAAGCAAAACTCTGTAAT ATTTCGCGGGATCGA	Reverse primer for promoter P65
P66f	TTAGATGAACTCTCCTTGACAAAATTTCCTATTAGGGGTATAATACCCCG TTTTGTTTAACTTTA	Forward primer for promoter P66
P66r	CGGGGTATTATACCCCTAATAGGAAATTTTGTCAAGGAGAGTTCATCTAA ATTTCGCGGGATCGA	Reverse primer for promoter P66
P67f	TCTCTCACGTGTAACTTGACAGTTGGTCTAGTGGTCGATATAATCCGGGG TTTTGTTTAACTTTA	Forward primer for promoter P67
P67r	CCCCGGATTATATCGACCACTAGACCAACTGTCAAGTTACACGTGAGAG AATTTCGCGGGATCGA	Reverse primer for promoter P67
P68f	TACGGAAGTCGGGACTTGACAAAAGAGGACTTATGCGCTATAATGAAAT TTTTTGTTTAACTTTA	Forward primer for promoter P68
P68r	AATTTCATTATAGCGCATAAGTCCTCTTTTGTCAAGTCCCGACTTCCGTA ATTTCGCGGGATCGA	Reverse primer for promoter P68
P69f	TGCGGTCTAATACCCTTGACAAAATACGCAATAGTATTATAATACCGTCC TTTTGTTTAACTTTA	Forward primer for promoter P69
P69r	GGACGGTATTATAATACTATTGCGTATTTTGTCAAGGGTATTAGACCGCA ATTTCGCGGGATCGA	Reverse primer for promoter P69
P70f	ATGTAACTTTGGAAGTTGACAAAAGGGACAGTTAGTGTATAATCTGCCT ATTTTGTTTAACTTTA	Forward primer for promoter P70
P70r	TAGGCAGATTATACACTAACTGTCCCTTTTGTCAACTTCCAAAGTTACAT ATTTCGCGGGATCGA	Reverse primer for promoter P70
P71f	TCGTGTATCTAGGGGTTGACAAAAGTCTCATGAGCGCCTATAATAGGCTA TTTTGTTTAACTTTA	Forward primer for promoter P71
P71r	TAGCCTATTATAGGCGCTCATGAGACTTTTGTCAACCCCTAGATACACGA ATTTCGCGGGATCGA	Reverse primer for promoter P71
P72f	CTACTCTTTGGTGATTGACAGGTCCAAGTGCCCGCCCCTATAATCATGCG	Forward primer for promoter P72

	TTTTGTTTAACTTTA	
P72r	CGCATGATTATAGGGGCGGCACTTGGACCTGTCAATCACCAAAGAGTAGATTTCGCGGGATCGA	Reverse primer for promoter P72
P73f	AATTCAGGATTCGACTTGACAAAATCATGTCAGTACGCTATAATAGCTCGTTTTGTTTAACTTTA	Forward primer for promoter P73
P73r	CGAGCTATTATAGCGTACTGACATGATTTTGTCAAGTCGAATCCTGAATTATTTCGCGGGATCGA	Reverse primer for promoter P73
P74f	TTTATCTTTCATTCCTTGACAATATTCTTCATTACTGCTATAATCCGGGGTTTTGTTTAACTTTA	Forward primer for promoter P74
P74r	CCCCGGATTATAGCAGTAATGAAGAATATTGTCAAGGAATGAAAGATAAAATTTCGCGGGATCGA	Reverse primer for promoter P74
P75f	ACTCTGATAGTTGGGTTGACAAAATACAGTAAGTGCATATAATAGGAATATTTTGTTTAACTTTA	Forward primer for promoter P75
P75r	TATTCCTATTATATGCACTTACTGTATTTTGTCAACCCAACTATCAGAGTATTTCGCGGGATCGA	Reverse primer for promoter P75
P76f	ATAATTAAAGCTGGCTTGACAATTGTATAACTCTATGGTATAATAGCGGCTTTTGTTTAACTTTA	Forward primer for promoter P76
P76r	GCCGCTATTATACCATAGAGTTATACAATTGTCAAGCCAGCTTTAATTATATTTCGCGGGATCGA	Reverse primer for promoter P76
P77f	AGCGGGATTTGACTCTTGACACAAGCGGGTAAACCACTATAATATACGCCTTTTGTTTAACTTTA	Forward primer for promoter P77
P77r	GGCGTATATTATAGTGGTTTACCCGCTTGTGTCAAGAGTCAAATCCCGCTATTTCGCGGGATCGA	Reverse primer for promoter P77
P78f	CTTTCAGTCGTTGCTTGACACAGTACGCTATACTCCAATATAATCCGGGGTTTTGTTTAACTTTA	Forward primer for promoter P78
P78r	CCCCGGATTATATTGGAGTATAGCGTACTGTGTCAAGCAACGACTGAAAGATTTCGCGGGATCGA	Reverse primer for promoter P78
P79f	TGCGGCGTAAGGGTTTTGACAAAAGTGTAACCATTCCGTATAATGGCCCCTTTTGTTTAACTTTA	Forward primer for promoter P79
P79r	GGGGCCATTATACGGAATGGTTACACTTTTGTCAAAACCCTTACGCCGCAATTTCGCGGGATCGA	Reverse primer for promoter P79
P80f	GGCGCGCAAGTCTAATTGACAAAAAAATAGCCGGTTGGTATAATTTGACGTTTTGTTTAACTTTA	Forward primer for promoter P80
P80r	CGTCAAATTATACCAACCGGCTATTTTTTTGTCAATTAGACTTGCGCGCCATTTCGCGGGATCGA	Reverse primer for promoter P80

P81f	GCTAGAAGTTAAACATTGACACAATCACTTAGCGCCTTATAATACTCTGTTTTTGTTTAACTTTA	Forward primer for promoter P81
P81r	ACAGAGTATTATAAGGCGCTAAGTGATTGTGTCAATGTTTAACTTCTAGCATTTCGCGGGATCGA	Reverse primer for promoter P81
P82f	CGCTGCAGCCTCTCCTTGACAAAATCGTAAAATGGGTCTATAATTAAGTATTTTGTTTAACTTTA	Forward primer for promoter P82
P82r	TACTTAATTATAGACCCATTTTACGATTTTGTCAAGGAGAGGCTGCAGCGATTTCGCGGGATCGA	Reverse primer for promoter P82
P83f	ACTAGTTAATTAGGCTTGACATTTGCGGTCCTATGGCCTATAATAGGTGGTTTTGTTTAACTTTA	Forward primer for promoter P83
P83r	CCACCTATTATAGGCCATAGGACCGCAAATGTCAAGCCTAATTAACTAGTATTTCGCGGGATCGA	Reverse primer for promoter P83
P84f	TAGAATTTATATGGCTTGACAAAAGAGTGGATTTCCGTATAATATAGTCTTTTTGTTTAACTTTA	Forward primer for promoter P84
P84r	AGACTATATTATACGGAAATCCACTCTTTTGTCAAGCCATATAAATTCTAATTTCGCGGGATCGA	Reverse primer for promoter P84
P85f	CATTGGTAAGAATGCTTGACAAAAGAAAAACTTAAACGTATAATGGGCCCTTTTGTTTAACTTTA	Forward primer for promoter P85
P85r	GGGCCCATTATACGTTTAAGTTTTTCTTTTGTCAAGCATTCTTACCAATGATTTCGCGGGATCGA	Reverse primer for promoter P85
P86f	TGTCTCTTACTCGCTTGACATGGTTGTCTATACGCGGCTATAATAACGCCTTTTGTTTAACTTTA	Forward primer for promoter P86
P86r	GGCGTTATTATAGCCGCGTATAGACAACCATGTCAAGCGAGTAAGAGACAATTTCGCGGGATCGA	Reverse primer for promoter P86
P87f	AATCAGATAAGTAAGTTGACAATAATTTTCAAATATGTATAATGGAGTCATTTTGTTTAACTTTA	Forward primer for promoter P87
P87r	TGACTCCATTATACATATTTGAAAATTATTGTCAACTTACTTATCTGATTATTTCGCGGGATCGA	Reverse primer for promoter P87
P88f	TTATCGAGATTCTGCTTGACAGAAGCCCGATTCTGAGGTATAATAAGCACTTTTGTTTAACTTTA	Forward primer for promoter P88
P88r	GTGCTTATTATACCTCAGAATCGGGCTTCTGTCAAGCAGAATCTCGATAAATTTCGCGGGATCGA	Reverse primer for promoter P88
P89f	ACCGCAAGGTTTGATTGACATGGTAGCGGCGCCTCACATATAATCCGGGCTTTTGTTTAACTTTA	Forward primer for promoter P89
P89r	GCCCGGATTATATGTGAGGCGCCGCTACCATGTCAATCAAACCTTGCGG	Reverse primer for promoter P89

	TATTTCGCGGGATCGA	
P90f	GACTATGCTTACGCTTGACACATTCTCTTACTCTCTGTTATAATCGCGGCTTTTGTTTAACTTTA	Forward primer for promoter P90
P90r	GCCGCGATTATAACAGAGAGTAAGAGAATGTGTCAAGCGTAAGCATAGTCATTTCGCGGGATCGA	Reverse primer for promoter P90
P91f	CGCCACCTAAATCTCTTGACATTTGGCCGTCAGCGGGCTATAATTTACCGTTTTGTTTAACTTTA	Forward primer for promoter P91
P91r	CGGTAAATTATAGCCCGCTGACGGCCAAATGTCAAGAGATTTAGGTGGCGATTTCGCGGGATCGA	Reverse primer for promoter P91
P92f	CTTCCTAAGCTCGAATTGACAGAACCTAAAATGAAGTTATAATAGGATATTTTTGTTTAACTTTA	Forward primer for promoter P92
P92r	ATATCCTATTATAACTTCATTTTAGGTTCTGTCAATTCGAGCTTAGGAAGATTTCGCGGGATCGA	Reverse primer for promoter P92
P93f	AGTAATCCGCCAACTTGACATGGTTGAAGATACGCCCGTATAATATAATTTTTTGTTTAACTTTA	Forward primer for promoter P93
P93r	AATTATATTATACGGGCGTATCTTCAACCATGTCAAGTTGGCGGATTACTATTTCGCGGGATCGA	Reverse primer for promoter P93
P94f	AGTTTTCAACGGGAATTGACACAACCAATTAATTTGTCTATAATATGCGTTTTTGTTTAACTTTA	Forward primer for promoter P94
P94r	ACGCATATTATAGACAAATTAATTGGTTGTGTCAATTCCCGTTGAAAACTATTTCGCGGGATCGA	Reverse primer for promoter P94
P95f	TTGGCACAATTTGGCTTGACAATAAATGAATTCTGAACTATAATCGCGGCTTTTGTTTAACTTTA	Forward primer for promoter P95
P95r	GCCGCGATTATAGTTCAGAATTCATTTATTGTCAAGCCAAATTGTGCCAAATTTCGCGGGATCGA	Reverse primer for promoter P95
P96f	ACTGGTCTAATAGTCTTGACATAAGAAAGATACTGGTCTATAATAGAGTTTTTTGTTTAACTTTA	Forward primer for promoter P96
P96r	AACTCTATTATAGACCAGTATCTTTCTTATGTCAAGACTATTAGACCAGTATTTCGCGGGATCGA	Reverse primer for promoter P96
P97f	TAATTTGCGCGACCTTGACAAGGTTGTATAGGCTTCCGTATAATTCAGAATTTTGTTTAACTTTA	Forward primer for promoter P97
P97r	TTCTGAATTATACGGAAGCCTATACAACCTTGTCAAGGTCGCGCAAATTAATTTCGCGGGATCGA	Reverse primer for promoter P97
P98f	TTAGAAAGGTTTACTTGACATGGTTCACATAATGGTGCTATAATTACTTATTTTGTTTAACTTTA	Forward primer for promoter P98

P98r	TAAGTAATTATAGCACCATTATGTGAACCATGTCAAGTAAACCTTTCTAAATTTCGCGGGATCGA	Reverse primer for promoter P98
P99f	CATATAAATGAAGATTGACATGTTCTTATATTAATTTCTATAATAGACGTTTTTGTTTAACTTTA	Forward primer for promoter P99
P99r	ACGTCTATTATAGAAATTAATATAAGAACATGTCAATCTTCATTTATATGATTTCGCGGGATCGA	Reverse primer for promoter P99
P100f	AGTCTCTTTCCCGACTTGACAAAAGATGTCGTATCCGGTATAATAGGTGGTTTTGTTTAACTTTA	Forward primer for promoter P100
P100r	CCACCTATTATACCGGATACGACATCTTTTGTCAAGTCGGGAAAGAGACTATTTCGCGGGATCGA	Reverse primer for promoter P100
P101f	TTGTGAGTTTCCAGCTTGACAAAACAGGCTATGAGTGTATAATAGCATCCTTTTGTTTAACTTTA	Forward primer for promoter P101
P101r	GGATGCTATTATACACTCATAGCCTGTTTTGTCAAGCTGGAAACTCACAAATTTCGCGGGATCGA	Reverse primer for promoter P101
P102f	TTGTGTTCAATTGTTTGACAAGGTTGCGTTTCCTTGCGTATAATGTGCCTTTTTGTTTAACTTTA	Forward primer for promoter P102
P102r	AGGCACATTATACGCAAGGAAACGCAACCTTGTCAAACAATTGAACACAAATTTCGCGGGATCGA	Reverse primer for promoter P102
P103f	ATTTTACCCATGTATTTGACAAAATTACCGCCCTCCGCTATAATGTACTTTTTTGTTTAACTTTA	Forward primer for promoter P103
P103r	AAGTACATTATAGCGGAGGGCGGTAATTTTGTCAAATACATGGGTAAAATATTTCGCGGGATCGA	Reverse primer for promoter P103
P104f	TATGATTCCTGTAGCTTGACAAAAGAGCTGTTAAATCCTATAATCCCCCATTTTGTTTAACTTTA	Forward primer for promoter P104
P104r	TGGGGGATTATAGGATTTAACAGCTCTTTTGTCAAGCTACAGGAATCATAATTTCGCGGGATCGA	Reverse primer for promoter P104
P105f	TGTGAAAGAGACGCCTTGACAAGAGTCGTCTCTGCCCTATAATAGGAATCTTTTGTTTAACTTTA	Forward primer for promoter P105
P105r	GATTCCTATTATAGGGCAGAGACGACTCTTGTCAAGGCGTCTCTTTCACAATTTCGCGGGATCGA	Reverse primer for promoter P105
P106f	TTTGATAAATAAGGCTTGACAAAAAACGCTACTCTCCTATAATACATATTTTTTGTTTAACTTTA	Forward primer for promoter P106
P106r	AATATGTATTATAGGAGAGTAGCGTTTTTTGTCAAGCCTTATTTATCAAAATTTCGCGGGATCGA	Reverse primer for promoter P106
P107f	AGCAGTGCGCGAACCTTGACAAAAGAAGTGCAAGGACGTATAATCTAG	Forward primer for promoter P107

	TGTTTTGTTTAACTTTA	
P107r	CACTAGATTATACGTCCTTGCACTTCTTTTGTCAAGGTTCGCGCACTGCTATTTCGCGGGATCGA	Reverse primer for promoter P107
P108f	TGGCACTTGAGGAGCTTGACAATAAAAGAACGGTTACTATAATAGCCCATTTTTGTTTAACTTTA	Forward primer for promoter P108
P108r	ATGGGCTATTATAGTAACCGTTCTTTTATTGTCAAGCTCCTCAAGTGCCAATTTCGCGGGATCGA	Reverse primer for promoter P108
P109f	ATGAGAAATGTTCGTTTGACAATAATAATTGTGGAGGGTATAATAGGTGGTTTTGTTTAACTTTA	Forward primer for promoter P109
P109r	CCACCTATTATACCCTCCACAATTATTATTGTCAAACGAACATTTCTCATATTTCGCGGGATCGA	Reverse primer for promoter P109
P110f	AAGTCTATAACGAACTTGACAATTGTTGCATTAGCGCTATAATATGCCATTTTTGTTTAACTTTA	Forward primer for promoter P110
P110r	ATGGCATATTATAGCGCTAATGCAACAATTGTCAAGTTCGTTATAGACTTATTTCGCGGGATCGA	Reverse primer for promoter P110
P111f	GTAGTTCCTTGTTCCTTGACAAAAGACTGTACTGGCGATATAATCCGGGGTTTTGTTTAACTTTA	Forward primer for promoter P111
P111r	CCCCGGATTATATCGCCAGTACAGTCTTTTGTCAAGGAACAAGGAACTACATTTCGCGGGATCGA	Reverse primer for promoter P111
P112f	GGCTAGAAGTCTCCCTTGACAAAATAATGTACAAGCGGTATAATTTGCAGTTTTGTTTAACTTTA	Forward primer for promoter P112
P112r	CTGCAAATTATACCGCTTGTACATTATTTTGTCAAGGGAGACTTCTAGCCATTTCGCGGGATCGA	Reverse primer for promoter P112
P113f	AAGCGGATGCGAATATTGACAGGTTCACCACAATAGGGTATAATAGGTGTTTTTGTTTAACTTTA	Forward primer for promoter P113
P113r	ACACCTATTATACCCTATTGTGGTGAACCTGTCAATATTCGCATCCGCTTATTTCGCGGGATCGA	Reverse primer for promoter P113
P114f	CTCACGTTTCCTACTTGACAAGGTTGCGTTTCCTTGAGTATAATGTGATATTTTGTTTAACTTTA	Forward primer for promoter P114
P114r	TATCACATTATACTCAAGGAAACGCAACCTTGTCAAGTAGGAAACGTGAGATTTCGCGGGATCGA	Reverse primer for promoter P114
P115f	TTAGTTGTGTCGTCATTGACAAAAGCCGCAAGCGCGGTATAATAGCACTATTTTGTTTAACTTTA	Forward primer for promoter P115
P115r	TAGTGCTATTATACCGCGCTTGCGGCTTTTGTCAATGACGACACAACTAAATTTCGCGGGATCGA	Reverse primer for promoter P115

P116f	AATGATTAAAGTAAATTGACAAAATTTTAATGTACACTATAATTATATCCTTTTGTTTAACTTTA	Forward primer for promoter P116
P116r	GGATATAATTATAGTGTACATTAAAATTTTGTCAATTTACTTTAATCATTATTTCGCGGGATCGA	Reverse primer for promoter P116
P117f	CACCCTGTTAATCCCTTGACAAAAGAAGGACTGTCATGTATAATAAGCCTTTTTGTTTAACTTTA	Forward primer for promoter P117
P117r	AGGCTTATTATACATGACAGTCCTTCTTTTGTCAAGGGATTAACAGGGTGATTTCGCGGGATCGA	Reverse primer for promoter P117
P118f	TCTGTAATAATTCGCTTGACAATTCCCGTAATGTAGCGTATAATAGGTCATTTTGTTTAACTTTA	Forward primer for promoter P118
P118r	TGACCTATTATACGCTACATTACGGGAATTGTCAAGCGAATTATTACAGAATTTCGCGGGATCGA	Reverse primer for promoter P118
P119f	AAAGAAGAGACCGCTTGACAGGTTCCTCTATACTAGGTTATAATTTGACTTTTTGTTTAACTTTA	Forward primer for promoter P119
P119r	AGTCAAATTATAACCTAGTATAGAGGAACCTGTCAAGCGGTCTCTTCTTTATTTCGCGGGATCGA	Reverse primer for promoter P119
P120f	TATCAGCAACTGGCTTGACAGCGTCGTAGTCAGGTTGATATAATAGGTGTTTTTGTTTAACTTTA	Forward primer for promoter P120
P120r	ACACCTATTATATCAACCTGACTACGACGCTGTCAAGCCAGTTGCTGATAATTTCGCGGGATCGA	Reverse primer for promoter P120
P121f	GCAATGAGCTCAACTTGACACATAGCGGAAATTTACTTTATAATAGCCCCTTTTGTTTAACTTTA	Forward primer for promoter P121
P121r	GGGGCTATTATAAAGTAAATTTCCGCTATGTGTCAAGTTGAGCTCATTGCATTTCGCGGGATCGA	Reverse primer for promoter P121
P122f	TTGAAAATAATCGATTGACATCGTTAACGATCCTTGCTTATAATTATTACTTTTGTTTAACTTTA	Forward primer for promoter P122
P122r	GTAATAATTATAAGCAAGGATCGTTAACGATGTCAATCGATTATTTTCAAATTTCGCGGGATCGA	Reverse primer for promoter P122
P123f	GAAGCTTGGTTGGATTGACAAGGTTTCCGCTCTGTTTTTATAATCTCCCCTTTTGTTTAACTTTA	Forward primer for promoter P123
P123r	GGGGAGATTATAAAAACAGAGCGGAAACCTTGTCAATCCAACCAAGCTTCATTTCGCGGGATCGA	Reverse primer for promoter P123
P124f	ATCCGAAGTTAGGATTGACACCTCCATGAGATTGGATCTATAATTAAAATTTTTGTTTAACTTTA	Forward primer for promoter P124
P124r	ATTTTAATTATAGATCCAATCTCATGGAGGTGTCAATCCTAACTTCGGATA	Reverse primer for promoter P124

	TTTCGCGGGATCGA	
P125f	CATGACCCACAATGTTTGACATAGGGCTTTAGGGTACTATAATAGGAATT<br>TTTTGTTTAACTTTA	Forward primer for promoter P125
P125r	AATTCCTATTATAGTACCCTAAAGCCCTATGTCAAACATTGTGGGTCATG<br>ATTTCGCGGGATCGA	Reverse primer for promoter P125
P126f	GCTGAGATTTAAGATTTGACAAAATTCGGCCTGCGAGGTATAATCAGTC<br>CTTTTGTTTAACTTTA	Forward primer for promoter P126
P126r	GGACTGATTATACCTCGCAGGCCGAATTTTGTCAAATCTTAAATCTCAGC<br>ATTTCGCGGGATCGA	Reverse primer for promoter P126
P127f	GTACTGTATCAAGATTGACATGGTTTACTATGTGGTTTTATAATAGCCCCT<br>TTTGTTTAACTTTA	Forward primer for promoter P127
P127r	GGGGCTATTATAAAACCACATAGTAAACCATGTCAATCTTGATACAGTAC<br>ATTTCGCGGGATCGA	Reverse primer for promoter P127
P128f	AAAAAAGGTTAACACTTGACAAAACAGTACGGGTACAGTATAATTGAC<br>GCTTTTGTTTAACTTTA	Forward primer for promoter P128
P128r	GCGTCAATTATACTGTACCCGTACTGTTTTGTCAAGTGTTAACCTTTTTTA<br>TTTCGCGGGATCGA	Reverse primer for promoter P128
P129f	TATGAGACCCCGGATTTGACAAAACAGCGTAAATGCCTATAATAGGACA<br>TTTTTGTTTAACTTTA	Forward primer for promoter P129
P129r	ATGTCCTATTATAGGCATTTACGCTGTTTTGTCAAATCCGGGGTCTCATAA<br>TTTCGCGGGATCGA	Reverse primer for promoter P129
P130f	AGCAGTCCCCCTAACTTGACATATTCGGTTATGTAGATTATAATAGCCCCT<br>TTTGTTTAACTTTA	Forward primer for promoter P130
P130r	GGGGCTATTATAATCTACATAACCGAATATGTCAAGTTAGGGGGACTGCT<br>ATTTCGCGGGATCGA	Reverse primer for promoter P130
P131f	ATGAAACTATGTGGCTTGACAAGAGTCCGTCTACGAGGTATAATCGATA<br>ATTTTGTTTAACTTTA	Forward primer for promoter P131
P131r	TTATCGATTATACCTCGTAGACGGACTCTTGTCAAGCCACATAGTTTCAT<br>ATTTCGCGGGATCGA	Reverse primer for promoter P131
P132f	AAGTCTCCCGTCGCTTGACATCGTCTGACCAACTCTAATATAATTAAAAC<br>TTTTGTTTAACTTTA	Forward primer for promoter P132
P132r	GTTTTAATTATATTAGAGTTGGTCAGACGATGTCAAGCGACGGGAGACT<br>TATTTCGCGGGATCGA	Reverse primer for promoter P132
P133f	GTAGCTACAGATCACTTGACAAAAGGACTTAGCTGTGTATAATTAGGCC<br>CTTTTGTTTAACTTTA	Forward primer for promoter P133

P133r	GGGCCTAATTATACACAGCTAAGTCCTTTTGTCAAGTGATCTGTAGCTAC ATTTCGCGGGATCGA	Reverse primer for promoter P133
P134f	ATGTGACTACAATGATTGACAAAAGGTGACCGACGGTCTATAATTATGT GTTTTGTTTAACTTTA	Forward primer for promoter P134
P134r	CACATAATTATAGACCGTCGGTCACCTTTTGTCAATCATTGTAGTCACAT ATTTCGCGGGATCGA	Reverse primer for promoter P134
P135f	ATGCCCTTATCATCCTTGACAAAAGAGTGGTAGGTGTCTATAATAGAAGT TTTTGTTTAACTTTA	Forward primer for promoter P135
P135r	ACTTCTATTATAGACACCTACCACTCTTTTGTCAAGGATGATAAGGGCAT ATTTCGCGGGATCGA	Reverse primer for promoter P135
P136f	TTCCTCTTACAATTCTTGACAAAAGCCACCCCCCGACTATAATAGGAAG TTTTTGTTTAACTTTA	Forward primer for promoter P136
P136r	ACTTCCTATTATAGTCGGGGGGTGGCTTTTGTCAAGAATTGTAAGAGGA AATTTCGCGGGATCGA	Reverse primer for promoter P136
P137f	ATCTAATCCAGATCCTTGACAAAAGTCTTCAATCCGAGTATAATTGTGCT TTTTGTTTAACTTTA	Forward primer for promoter P137
P137r	AGCACAATTATACTCGGATTGAAGACTTTTGTCAAGGATCTGGATTAGAT ATTTCGCGGGATCGA	Reverse primer for promoter P137
P138f	GAAATGACTGAGACTTGACATCGTAGCGTAGCCGTGGGTATAATGGAAC ATTTTGTTTAACTTTA	Forward primer for promoter P138
P138r	TGTTCCATTATACCCACGGCTACGCTACGATGTCAAGTCTCAGTCATTTC ATTTCGCGGGATCGA	Reverse primer for promoter P138
P139f	TCCCCCTTAATCCATTGACAAGGTTGTTAGATCTCGCGTATAATCCGCGC TTTTGTTTAACTTTA	Forward primer for promoter P139
P139r	GCGCGGATTATACGCGAGATCTAACAACCTTGTCAATGGATTAAGGGGG AATTTCGCGGGATCGA	Reverse primer for promoter P139
P140f	TATGAAAGTGACTCCTTGACAAAACGTCCTAAATGAGATATAATCGGTC GTTTTGTTTAACTTTA	Forward primer for promoter P140
P140r	CGACCGATTATATCTCATTTAGGACGTTTTGTCAAGGAGTCACTTTCATA ATTTCGCGGGATCGA	Reverse primer for promoter P140
P141f	AACTGTAAGACACCATTGACAAAAGTGCTCTCCGCGCTATAATAGAAAA ATTTTGTTTAACTTTA	Forward primer for promoter P141
P141r	TTTTTCTATTATAGCGCGGAGAGCACTTTTGTCAATGGTGTCTTACAGTT ATTTCGCGGGATCGA	Reverse primer for promoter P141
P142f	TTGTGGCGTCCCTCCTTGACAAAATACGATCCCCCGAGTATAATTGAATA	Forward primer for promoter P142

	TTTTGTTTAACTTTA	
P142r	TATTCAATTATACTCGGGGGATCGTATTTTGTCAAGGAGGGACGCCACAAATTTCGCGGGATCGA	Reverse primer for promoter P142
P143f	CCTAGCCGGGGTGAATTGACAGAAGTCAGGAACAGAGTATAATAGACTACTTTTGTTTAACTTTA	Forward primer for promoter P143
P143r	GTAGTCTATTATACTCTGTTCCTGACTTCTGTCAATTCACCCCGGCTAGGATTTCGCGGGATCGA	Reverse primer for promoter P143
P144f	TCGATAGGTGTGGCCTTGACAGAAGGTGTATAAAGGGTATAATAGCACCCTTTTGTTTAACTTTA	Forward primer for promoter P144
P144r	GGGTGCTATTATACCCTTTATACACCTTCTGTCAAGGCCACACCTATCGAATTTCGCGGGATCGA	Reverse primer for promoter P144
P145f	ATGTCATTGTGTCCTTGACAAGGTTCTGCATAGGTGTTTATAATCGGTGTTTTTGTTTAACTTTA	Forward primer for promoter P145
P145r	ACACCGATTATAAACACCTATGCAGAACCTTGTCAAGGACACAATGACATATTTCGCGGGATCGA	Reverse primer for promoter P145
P146f	CTCTCTGAATATACTTGACAAGGTAAGGTGTTGAACTTTATAATAGCGCCTTTTGTTTAACTTTA	Forward primer for promoter P146
P146r	GGCGCTATTATAAAGTTCAACACCTTACCTTGTCAAGTATATTCAGAGAGATTTCGCGGGATCGA	Reverse primer for promoter P146
P147f	GCACGCTGGTTTATTTGACAGGATAAAAACTCTCATATTATAATAGGCAATTTTGTTTAACTTTA	Forward primer for promoter P147
P147r	TTGCCTATTATAATATGAGAGTTTTTATCCTGTCAAATAAACCAGCGTGCATTTCGCGGGATCGA	Reverse primer for promoter P147
P148f	CGTAAGGGGCTTGCTTGACATCGTTACTAGCAAGAAGCTATAATCTAGCATTTTGTTTAACTTTA	Forward primer for promoter P148
P148r	TGCTAGATTATAGCTTCTTGCTAGTAACGATGTCAAGCAAGCCCCTTACGATTTCGCGGGATCGA	Reverse primer for promoter P148
P149f	CTCGTTAGCTAATTTTGACAAGGTGACTTATACTATGATATAATTCACGGTTTTGTTTAACTTTA	Forward primer for promoter P149
P149r	CCGTGAATTATATCATAGTATAAGTCACCTTGTCAAAATTAGCTAACGAGATTTCGCGGGATCGA	Reverse primer for promoter P149
P150f	AGTGAAACATATAGTTGACACAAACCCCTGGCAGTGGTTATAATCGAGCATTTTGTTTAACTTTA	Forward primer for promoter P150
P150r	TGCTCGATTATAACCACTGCCAGGGGTTTGTGTCAACTATATGTTTCACTATTTCGCGGGATCGA	Reverse primer for promoter P150

P151f	TGTACGCTTGACCACTTGACAGAAGAGTAGTGTGTGCATATAATTTGGAGTTTTGTTTAACTTTA	Forward primer for promoter P151
P151r	CTCCAAATTATATGCACACACTACTCTTCTGTCAAGTGGTCAAGCGTACAATTTCGCGGGATCGA	Reverse primer for promoter P151
P152f	AGCTAACCCGTTCCCTTGACAAAATTACGAATATACACTATAATCCCGCATTTTGTTTAACTTTA	Forward primer for promoter P152
P152r	TGCGGGATTATAGTGTATATTCGTAATTTTGTCAAGGGAACGGGTTAGCTATTTCGCGGGATCGA	Reverse primer for promoter P152
P153f	ACAAACTCTTGCTGCTTGACAATAATATCAGCCTTGGTATAATAGAATCATTTTGTTTAACTTTA	Forward primer for promoter P153
P153r	TGATTCTATTATACCAAGGCTGATATTATTGTCAAGCAGCAAGAGTTTGTATTTCGCGGGATCGA	Reverse primer for promoter P153
P154f	GTTACAAATTCTGAATTGACAGAAGAGTGGGCGACTCATATAATGATACGTTTTGTTTAACTTTA	Forward primer for promoter P154
P154r	CGTATCATTATATGAGTCGCCCACTCTTCTGTCAATTCAGAATTTGTAACATTTCGCGGGATCGA	Reverse primer for promoter P154
P155f	ACTATGTTTATCTCTTTGACAGAACGGGCAATATGGGTATAATAAGAATTTTTTGTTTAACTTTA	Forward primer for promoter P155
P155r	AATTCTTATTATACCCATATTGCCCGTTCTGTCAAAGAGATAAACATAGTATTTCGCGGGATCGA	Reverse primer for promoter P155
P156f	GGGAACCTAAATACTTGACAATGTTGCCGGTCGGTGGTTATAATGAGTAATTTTGTTTAACTTTA	Forward primer for promoter P156
P156r	TTACTCATTATAACCACCGACCGGCAACATTGTCAAGTATTTAGGTTCCCATTTCGCGGGATCGA	Reverse primer for promoter P156
P157f	AAAAAAAGGTGTACCTTGACAGTTGCAAAACTTTGGGTTATAATGTCGCATTTTGTTTAACTTTA	Forward primer for promoter P157
P157r	TGCGACATTATAACCCAAAGTTTTGCAACTGTCAAGGTACACCTTTTTTTATTTCGCGGGATCGA	Reverse primer for promoter P157
P158f	TGCGCAAAGATCGATTGACATCAGTTCCTAGTAGAAAATATAATTACCCCTTTTGTTTAACTTTA	Forward primer for promoter P158
P158r	GGGGTAATTATATTTTCTACTAGGAACTGATGTCAATCGATCTTTGCGCAATTTCGCGGGATCGA	Reverse primer for promoter P158
P159f	AAATCTTTACCATCTTGACACTTAATCCAATCCGTTTTTATAATCGCGGCTTTTGTTTAACTTTA	Forward primer for promoter P159
P159r	GCCGCGATTATAAAAACGGATTGGATTAAGTGTCAAGATGGTAAAGATT	Reverse primer for promoter P159

	TATTTCGCGGGATCGA	
P160f	TTAGTCGTTCGAGCCTTGACAAGGTCGCTCCTAAAGGCTATAATCGGGG GTTTTGTTTAACTTTA	Forward primer for promoter P160
P160r	CCCCCGATTATAGCCTTTAGGAGCGACCTTGTCAAGGCTCGAACGACTA AATTTCGCGGGATCGA	Reverse primer for promoter P160
P161f	TATGAAACTTAGGGCTTGACATATGCGGCGTCGTTTGTTATAATTGAACA TTTTGTTTAACTTTA	Forward primer for promoter P161
P161r	TGTTCAATTATAACAAACGACGCCGCATATGTCAAGCCCTAAGTTTCATA ATTTCGCGGGATCGA	Reverse primer for promoter P161
P162f	CGAATGTGGACTTCGTTGACAAAAGACACGCAAGATGTTATAATATAGC ATTTTGTTTAACTTTA	Forward primer for promoter P162
P162r	TGCTATATTATAACATCTTGCGTGTCTTTTGTCAACGAAGTCCACATTCG ATTTCGCGGGATCGA	Reverse primer for promoter P162
P163f	CGTAAACCTCGCGATTGACACTGTAAGATAGTACTCGTTATAATTGAGCA TTTTGTTTAACTTTA	Forward primer for promoter P163
P163r	TGCTCAATTATAACGAGTACTATCTTACAGTGTCAATCGCGAGGTTTACG ATTTCGCGGGATCGA	Reverse primer for promoter P163
P164f	ACGTCGTTGGGCACCTTGACAAAATGGGCTATGCTGGTATAATGGCAAC TTTTTGTTTAACTTTA	Forward primer for promoter P164
P164r	AGTTGCCATTATACCAGCATAGCCCATTTTGTCAAGGTGCCCAACGACG TATTTCGCGGGATCGA	Reverse primer for promoter P164
P165f	AGCCCGCTAGAGGGTTTGACAGTTGAAGCATACTATGGTATAATCGATG ATTTTGTTTAACTTTA	Forward primer for promoter P165
P165r	TCATCGATTATACCATAGTATGCTTCAACTGTCAAACCCTCTAGCGGGCT ATTTCGCGGGATCGA	Reverse primer for promoter P165
P166f	CGCATGTCTCTAAATTGACAGGATAATATAGGTACTGGTATAATATGCGAT TTTGTTTAACTTTA	Forward primer for promoter P166
P166r	TCGCATATTATACCAGTACCTATATTATCCTGTCAATTTAGAGACATGCGA TTTCGCGGGATCGA	Reverse primer for promoter P166
P167f	CTGGCACCTGTTCCTTGACACATACGAAAACTGAGCAATATAATATCCGT TTTTGTTTAACTTTA	Forward primer for promoter P167
P167r	ACGGATATTATATTGCTCAGTTTTCGTATGTGTCAAGGAACAGGTGCCAG ATTTCGCGGGATCGA	Reverse primer for promoter P167
P168f	TAGTCTCCCCCTAATTGACATTAGTCGAGATAAGAACGTATAATGCTTAG TTTTGTTTAACTTTA	Forward primer for promoter P168

P168r	CTAAGCATTATACGTTCTTATCTCGACTAATGTCAATTAGGGGGAGACTAATTTCGCGGGATCGA	Reverse primer for promoter P168
P169f	TTCTACTCCGCGGGGTTGACAATTGCTTCGTGTGTGTTTATAATCGCGCGTTTTGTTTAACTTTA	Forward primer for promoter P169
P169r	CGCGCGATTATAAACACACACGAAGCAATTGTCAACCCCGCGGAGTAGAAATTTCGCGGGATCGA	Reverse primer for promoter P169
P170f	CCTAAACTATACTGCTTGACAATATGTATCACGGATGTATAATAGGAATTTTTTGTTTAACTTTA	Forward primer for promoter P170
P170r	AATTCCTATTATACATCCGTGATACATATTGTCAAGCAGTATAGTTTAGGATTTCGCGGGATCGA	Reverse primer for promoter P170
P171f	GTCGGAAGCTATTCTTGACAGCATACATTTAAGCATGTTATAATCCAGGATTTTGTTTAACTTTA	Forward primer for promoter P171
P171r	TCCTGGATTATAACATGCTTAAATGTATGCTGTCAAGAATAGCTTCCGACATTTCGCGGGATCGA	Reverse primer for promoter P171
P172f	AGAGGTACGTCCGATTGACATTAGACATAAGTGAGGTTTATAATAGCCGATTTTGTTTAACTTTA	Forward primer for promoter P172
P172r	TCGGCTATTATAAACCTCACTTATGTCTAATGTCAATCGGACGTACCTCTATTTCGCGGGATCGA	Reverse primer for promoter P172
P173f	AAAATCTGTTGTGATTGACATTCTTCAAGTAAGTATGGTATAATTTATGATTTTGTTTAACTTTA	Forward primer for promoter P173
P173r	TCATAAATTATACCATACTTACTTGAAGAATGTCAATCACAACAGATTTTATTTCGCGGGATCGA	Reverse primer for promoter P173
P174f	TCGGTGAGTCGCAATTGACAAGGTTCGTAAAGGTTACGTATAATCAGGAGTTTTGTTTAACTTTA	Forward primer for promoter P174
P174r	CTCCTGATTATACGTAACCTTTACGAACCTTGTCAATTGCGACTCACCGAATTTCGCGGGATCGA	Reverse primer for promoter P174
P175f	CATCAACCTCTGTACTTGACAAAAGATTAACTTATTTCTATAATAGACGCTTTTGTTTAACTTTA	Forward primer for promoter P175
P175r	GCGTCTATTATAGAAATAAGTTAATCTTTTGTCAAGTACAGAGGTTGATGATTTCGCGGGATCGA	Reverse primer for promoter P175
P176f	CCACACTTTGTACATTGACAAAAAAAAATACAATCACTTATAATCGCGGATTTTGTTTAACTTTA	Forward primer for promoter P176
P176r	TCCGCGATTATAAGTGATTGTATTTTTTTTTGTCAATGTACAAAGTGTGGATTTCGCGGGATCGA	Reverse primer for promoter P176
P177f	ATTTAAAAAAAACGCTTGACAGAACAACGTTACTCACCTATAATCTGAA	Forward primer for promoter P177

	GTTTTGTTTAACTTTA	
P177r	CTTCAGATTATAGGTGAGTAACGTTGTTCTGTCAAGCGTTTTTTTAAAT<br>ATTTCGCGGGATCGA	Reverse primer for promoter P177
P178f	ACCATGTACCCGGACTTGACAAAAGAGGGACAATTCGTATAATAGGACT<br>TTTTTGTTTAACTTTA	Forward primer for promoter P178
P178r	AAGTCCTATTATACGAATTGTCCCTCTTTTGTCAAGTCCGGGTACATGGT<br>ATTTCGCGGGATCGA	Reverse primer for promoter P178
P179f	ATGCAATAATGTCCATTGACAAAAGCTGGTATCCCCCTATAATACGTCCC<br>TTTTGTTTAACTTTA	Forward primer for promoter P179
P179r	GGGACGTATTATAGGGGGATACCAGCTTTTGTCAATGGACATTATTGCAT<br>ATTTCGCGGGATCGA	Reverse primer for promoter P179
P180f	TGTGTAACTAAGCCTTGACAAGGTTTATCATACTTGTTTATAATCGAACA<br>TTTTGTTTAACTTTA	Forward primer for promoter P180
P180r	TGTTCGATTATAAACAAGTATGATAAACCTTGTCAAGGCTTAGTTACACA<br>ATTTCGCGGGATCGA	Reverse primer for promoter P180
P181f	AGTGATTCTGACTTATTGACACAAGAAATCGAACTGAGTATAATAGCCT<br>GTTTTGTTTAACTTTA	Forward primer for promoter P181
P181r	CAGGCTATTATACTCAGTTCGATTTCTTGTGTCAATAAGTCAGAATCACT<br>ATTTCGCGGGATCGA	Reverse primer for promoter P181
P182f	GGTTACCCGCGAAGGTTGACAAAATTTATTTGGTACGTATAATAGAATCT<br>TTTTGTTTAACTTTA	Forward primer for promoter P182
P182r	AGATTCTATTATACGTACCAAATAAATTTTGTCAACCTTCGCGGGTAACC<br>ATTTCGCGGGATCGA	Reverse primer for promoter P182
P183f	GCTCCGGATCTAGATTGACAGCTGCGGGGCTAATGTGGTATAATCCAGC<br>ATTTTGTTTAACTTTA	Forward primer for promoter P183
P183r	TGCTGGATTATACCACATTAGCCCCGCAGCTGTCAATCTAGATCCGGAGC<br>ATTTCGCGGGATCGA	Reverse primer for promoter P183
P184f	AATGACGCTCGAGATTGACATTCTCGAACCCTCTAGGTTATAATTCTCTC<br>TTTTGTTTAACTTTA	Forward primer for promoter P184
P184r	GAGAGAATTATAACCTAGAGGGTTCGAGAATGTCAATCTCGAGCGTCAT<br>TATTTCGCGGGATCGA	Reverse primer for promoter P184
P185f	AGTTGACGTCGTTAGTTGACAGTTGCGATGGCTAACCTATAATAGACTA<br>GTTTTGTTTAACTTTA	Forward primer for promoter P185
P185r	CTAGTCTATTATAGGTTAGCCATCGCAACTGTCAACTAACGACGTCAACT<br>ATTTCGCGGGATCGA	Reverse primer for promoter P185

P186f	ACAGTCGTGAGTCAATTGACAAAATATTAAGAGTCCCTATAATGGACTAGTTTTGTTTAACTTTA	Forward primer for promoter P186
P186r	CTAGTCCATTATAGGGACTCTTAATATTTTGTCAATTGACTCACGACTGTATTTCGCGGGATCGA	Reverse primer for promoter P186
P187f	AGTCGAACGAGGACCTTGACAAAAGGTATTAAGGTACTATAATAGGAATGTTTTGTTTAACTTTA	Forward primer for promoter P187
P187r	CATTCCTATTATAGTACCTTAATACCTTTTGTCAAGGTCCTCGTTCGACTATTTCGCGGGATCGA	Reverse primer for promoter P187
P188f	GATTAGACTTAGTTATTGACACATAGGAAACACGTACTATAATATCCTACTTTTGTTTAACTTTA	Forward primer for promoter P188
P188r	GTAGGATATTATAGTACGTGTTTCCTATGTGTCAATAACTAAGTCTAATCATTTCGCGGGATCGA	Reverse primer for promoter P188
P189f	AAGGAATAGTTTCCCTTGACAAAATCTAGACTTTAAATATAATAGCACGATTTTGTTTAACTTTA	Forward primer for promoter P189
P189r	TCGTGCTATTATATTTAAAGTCTAGATTTTGTCAAGGGAAACTATTCCTTATTTCGCGGGATCGA	Reverse primer for promoter P189
P190f	ACATCTAAGTCTGGCTTGACAAGACCCGTATACGTTTTATAATATACTCTTTTTGTTTAACTTTA	Forward primer for promoter P190
P190r	AGAGTATATTATAAAACGTATACGGGTCTTGTCAAGCCAGACTTAGATGTATTTCGCGGGATCGA	Reverse primer for promoter P190
P191f	TATGTTCTAATATTATTGACAGAAGCTGATTGGTGATTATAATTTGTTATTTTTGTTTAACTTTA	Forward primer for promoter P191
P191r	ATAACAAATTATAATCACCAATCAGCTTCTGTCAATAATATTAGAACATAATTTCGCGGGATCGA	Reverse primer for promoter P191
P192f	CGTCAAACCAGTGGCTTGACAAAAGCAAGTGCTGCGCTATAATCACGCTCTTTTGTTTAACTTTA	Forward primer for promoter P192
P192r	GAGCGTGATTATAGCGCAGCACTTGCTTTTGTCAAGCCACTGGTTTGACGATTTCGCGGGATCGA	Reverse primer for promoter P192
P193f	TATGAACTAAAGAGATTGACAAAATGCCATGAGACCTTATAATACGCTATTTTTGTTTAACTTTA	Forward primer for promoter P193
P193r	ATAGCGTATTATAAGGTCTCATGGCATTTTGTCAATCTCTTTAGTTCATAATTTCGCGGGATCGA	Reverse primer for promoter P193
P194f	CATAACAAGAAAGATTGACATTTTCATAAACAATAGCCTATAATATGTTATTTTGTTTAACTTTA	Forward primer for promoter P194
P194r	TAACATATTATAGGCTATTGTTTATGAAAATGTCAATCTTTCTTGTTATGAT	Reverse primer for promoter P194

	TTCGCGGGATCGA	
P195f	TTTCAAACATCATCCTTGACAGAAGGTGTATTTAGTGTATAATTTTTACATTTTGTTTAACTTTA	Forward primer for promoter P195
P195r	TGTAAAAATTATACACTAAATACACCTTCTGTCAAGGATGATGTTTGAAAATTTCGCGGGATCGA	Reverse primer for promoter P195
P196f	ATCTTGATTTGTACTTGACACAGACATGTGTTGGCACTTATAATCACCGTTTTTGTTTAACTTTA	Forward primer for promoter P196
P196r	ACGGTGATTATAAGTGCCAACACATGTCTGTGTCAAGTACAAATCAAGATATTTCGCGGGATCGA	Reverse primer for promoter P196
P197f	GTCCGGTTCCCCCCCTTGACAGTTGGTGGCGATCTGAGTATAATATCCGTTTTTGTTTAACTTTA	Forward primer for promoter P197
P197r	ACGGATATTATACTCAGATCGCCACCAACTGTCAAGGGGGGGAACCGGACATTTCGCGGGATCGA	Reverse primer for promoter P197
P198f	CCTTACAGACTCTCCTTGACAGAAGTGCTGATAATTCTATAATGGCTGTTTTTTGTTTAACTTTA	Forward primer for promoter P198
P198r	AACAGCCATTATAGAATTATCAGCACTTCTGTCAAGGAGAGTCTGTAAGGATTTCGCGGGATCGA	Reverse primer for promoter P198
P199f	CCTGAAATTTGAGCCTTGACATTAATCAGTAAGTACAGTATAATGCGCGCTTTTGTTTAACTTTA	Forward primer for promoter P199
P199r	GCGCGCATTATACTGTACTTACTGATTAATGTCAAGGCTCAAATTTCAGGATTTCGCGGGATCGA	Reverse primer for promoter P199
P200f	GTCGTAAGTCTTGATTGACATTCTCATCACTGCACAGATATAATCGCGGCTTTTGTTTAACTTTA	Forward primer for promoter P200
P200r	GCCGCGATTATATCTGTGCAGTGATGAGAATGTCAATCAAGACTTACGACATTTCGCGGGATCGA	Reverse primer for promoter P200
P1f	CCTACTAATTCTTGCTTGACATAAGCGATATAACCTGTATAATTAGACTGTTTTGTTTAACTTTA	Forward primer for promoter S1
P1r	CAGTCTAATTATACAGGTTATATCGCTTATGTCAAGCAAGAATTAGTAGGATTTCGCGGGATCGA	Reverse primer for promoter S1
P2f	TTTGAAACCAGTCAATTGACAGAAGAAAACTCGGCTTTATAATAGAGTGATTTTGTTTAACTTTA	Forward primer for promoter S2
P2r	TCACTCTATTATAAAGCCGAGTTTTCTTCTGTCAATTGACTGGTTTCAAAATTTCGCGGGATCGA	Reverse primer for promoter S2
P3f	CATGAGGTTCTTGGGTTGACAGAACCTCATCGTGGGCTATAATAGCACTCTTTTGTTTAACTTTA	Forward primer for promoter S3

P3r	GAGTGCTATTATAGCCCACGATGAGGTTCTGTCAACCCAAGAACCTCATGATTTCGCGGGATCGA	Reverse primer for promoter S3
P4f	ACGCTCCCCGTTACTTGACATTATTAGGAATTTGAACTTATAATAGGGCGTTTTGTTTAACTTTA	Forward primer for promoter S4
P4r	CGCCCTATTATAAGTTCAAATTCCTAATAATGTCAAGTAACGGGGAGCGTATTTCGCGGGATCGA	Reverse primer for promoter S4
P5f	AGATTAGGATAACCATTGACAAAAGCTCTATTCTCTGTATAATGTGGGGGTTTTGTTTAACTTTA	Forward primer for promoter S5
P5r	CCCCCACATTATACAGAGAATAGAGCTTTTGTCAATGGTTATCCTAATCTATTTCGCGGGATCGA	Reverse primer for promoter S5
P6f	CTGCCCTTAGGTTGGTTGACAAAAGCAAACACATTTTTATAATACATTCATTTTGTTTAACTTTA	Forward primer for promoter S6
P6r	TGAATGTATTATAAAAATGTGTTTGCTTTTGTCAACCAACCTAAGGGCAGATTTCGCGGGATCGA	Reverse primer for promoter S6
P7f	CGGCAAGCAGACTCCTTGACAATAAGCGCGTCATGAATATAATACACTCGTTTTGTTTAACTTTA	Forward primer for promoter S7
P7r	CGAGTGTATTATATTCATGACGCGCTTATTGTCAAGGAGTCTGCTTGCCGATTTCGCGGGATCGA	Reverse primer for promoter S7
P8f	GAAATCTTTGAAGGCTTGACAAAAGCGACGTGTGAGTTTATAATCCTGATTTTTGTTTAACTTTA	Forward primer for promoter S8
P8r	ATCAGGATTATAAACTCACACGTCGCTTTTGTCAAGCCTTCAAAGATTTCATTTCGCGGGATCGA	Reverse primer for promoter S8
P9f	AAAAAAATAATCCCTTGACAAGGTTATCTCCCCTAGTATATAATTAATTGTTTTGTTTAACTTTA	Forward primer for promoter S9
P9r	CAATTAATTATATACTAGGGGAGATAACCTTGTCAAGGGATTATTTTTTTATTTCGCGGGATCGA	Reverse primer for promoter S9
P10f	TGCGGTCTAATACCCTTGACAAAATACGCAATAGTATTATAATACCGTCCTTTTGTTTAACTTTA	Forward primer for promoter S10
P10r	GGACGGTATTATAATACTATTGCGTATTTTGTCAAGGGTATTAGACCGCAATTTCGCGGGATCGA	Reverse primer for promoter S10
P11f	CATAGCCTAGTATCTTGACAAGGTTATAGTCCGCACCCTATAATCTGATTTTTTGTTTAACTTTA	Forward primer for promoter S11
P11r	AATCAGATTATAGGGTGCGGACTATAACCTTGTCAAGATACTAGGCTATGATTTCGCGGGATCGA	Reverse primer for promoter S11
P12f	GAGTGAGTCCGGTTCTTGACAGAAGACTATGAAAAGCTATAATAGCCTA	Forward primer for promoter S12

	GTTTTGTTTAACTTTA	
P12r	CTAGGCTATTATAGCTTTTCATAGTCTTCTGTCAAGAACCGGACTCACTC ATTTCGCGGGATCGA	Reverse primer for promoter S12
P13f	CGACTCACATCATCGTTGACATTTGCTTATTGGTATTTATAATATACTAATT TTGTTTAACTTTA	Forward primer for promoter S13
P13r	TTAGTATATTATAAATACCAATAAGCAAATGTCAACGATGATGTGAGTCG ATTTCGCGGGATCGA	Reverse primer for promoter S13
P14f	TATTACTTCGCCTATTTGACAAAAGAGGTTAAGGTTGTATAATAGTACCA TTTTGTTTAACTTTA	Forward primer for promoter S14
P14r	TGGTACTATTATACAACCTTAACCTCTTTTGTCAAATAGGCGAAGTAATA ATTTCGCGGGATCGA	Reverse primer for promoter S14
P15f	GACCGCGCGAGCGATTGACATCGTAATGTAATTCCGCGTATAATAATGTG TTTTGTTTAACTTTA	Forward primer for promoter S15
P15r	CACATTATTATACGCGGAATTACATTACGATGTCAATCGCTCGCGCGGTC ATTTCGCGGGATCGA	Reverse primer for promoter S15
P16f	TGTGAAACTTATCCGTTGACAAGATAACTAATTAGTATATAATACATATTT TTTGTTTAACTTTA	Forward primer for promoter S16
P16r	AATATGTATTATATACTAATTAGTTATCTTGTCAACGGATAAGTTTCACAA TTTCGCGGGATCGA	Reverse primer for promoter S16
P17f	GCTAACCTAATGGATTGACATCAGTAGGCCAGGTACTATATAATGACACG TTTTGTTTAACTTTA	Forward primer for promoter S17
P17r	CGTGTCATTATATAGTACCTGGCCTACTGATGTCAATCCATTAGGTTAGCA TTTCGCGGGATCGA	Reverse primer for promoter S17
P18f	AAGTGGGTACTGTCCTTGACAAAAGATAAACAAGAGGGTATAATGAAC CATTTTGTTTAACTTTA	Forward primer for promoter S18
P18r	TGGTTCATTATACCCTCTTGTTTATCTTTTGTCAAGGACAGTACCCACTTA TTTCGCGGGATCGA	Reverse primer for promoter S18
P19f	ACATCTTGACCAACCTTGACAAAAGCCGTATAAGAACGTATAATTTCTC GTTTTGTTTAACTTTA	Forward primer for promoter S19
P19r	CGAGAAATTATACGTTCTTATACGGCTTTTGTCAAGGTTGGTCAAGATGT ATTTCGCGGGATCGA	Reverse primer for promoter S19
P20f	AGCGAGAGTTAAGATTGACAGCGACTTCCTAAATGGTCTATAATTTGCA CTTTTGTTTAACTTTA	Forward primer for promoter S20
P20r	GTGCAAATTATAGACCATTTAGGAAGTCGCTGTCAATCTTAACTCTCGCT ATTTCGCGGGATCGA	Reverse primer for promoter S20

P21f	ATCTTAAATTGTTCATTGACAGTAAGGTAAAAATTGAGTATAATAGCCCTTTTTGTTTAACTTTA	Forward primer for promoter S21
P21r	AGGGCTATTATACTCAATTTTTACCTTACTGTCAATGAACAATTTAAGATATTTCGCGGGATCGA	Reverse primer for promoter S21
P22f	TATACTATCTAACACTTGACAAAATGAGACGTGTATATATAATTAGGTGGTTTTGTTTAACTTTA	Forward primer for promoter S22
P22r	CCACCTAATTATATATACACGTCTCATTTTGTCAAGTGTTAGATAGTATAATTTCGCGGGATCGA	Reverse primer for promoter S22
P23f	CTTATATTAGAGTCATTGACAGTTGTCAATAGGTCAGTTATAATAGGTACTTTTGTTTAACTTTA	Forward primer for promoter S23
P23r	GTACCTATTATAACTGACCTATTGACAACTGTCAATGACTCTAATATAAGATTTCGCGGGATCGA	Reverse primer for promoter S23
P24f	TTGTCTATAACAGATTGACATCATATTTCAATCTCTGTTATAATTGTGAGTTTTGTTTAACTTTA	Forward primer for promoter S24
P24r	CTCACAATTATAACAGAGATTGAAATATGATGTCAATCTGTTATAGACAAATTTCGCGGGATCGA	Reverse primer for promoter S24
P25f	AGTATAGATAGTTGCTTGACAAAAGTCAAACTGGACAGTATAATTATTCATTTTGTTTAACTTTA	Forward primer for promoter S25
P25r	TGAATAATTATACTGTCCAGTTTGACTTTTGTCAAGCAACTATCTATACTATTTCGCGGGATCGA	Reverse primer for promoter S25
P26f	TATGACACTTAGAATTGACATCAACCCTCCAGTTTGGTTATAATTTGGCTTTTTGTTTAACTTTA	Forward primer for promoter S26
P26r	AGCCAAATTATAACCAAACTGGAGGGTTGATGTCAATTCTAAGTGTCATAATTTCGCGGGATCGA	Reverse primer for promoter S26
P27f	CATAACTTGTGCGATTGACAAGGTTCATCAAGTCCCCGTATAATTAACCTTTTTGTTTAACTTTA	Forward primer for promoter S27
P27r	AGGTTAATTATACGGGGACTTGATGAACCTTGTCAATCGCACAAGTTATGATTTCGCGGGATCGA	Reverse primer for promoter S27
P28f	AAAATACTAACCATTTGACATCGTTATGTAGAGGTAACTATAATCCGGGATTTTGTTTAACTTTA	Forward primer for promoter S28
P28r	TCCCGGATTATAGTTACCTCTACATAACGATGTCAAATGGTTAGTATTTTATTTCGCGGGATCGA	Reverse primer for promoter S28
P29f	AGAATTATGAAGGGCTTGACAAAATGGGCGTGTACTTTATAATTATACCTTTTTGTTTAACTTTA	Forward primer for promoter S29
P29r	AGGTATAATTATAAAGTACACGCCCATTTTGTCAAGCCCTTCATAATTCTA	Reverse primer for promoter S29

	TTTCGCGGATCGA	
P30f	TCTTCTATGATCAATTGACAAGGTGACGGAAATCCCTTTATAATGCCCCCTTTTGTTTAACTTTA	Forward primer for promoter S30
P30r	GGGGGCATTATAAAGGGATTTCCGTCACCTTGTCAATTGATCATAGAAGAATTTCGCGGGATCGA	Reverse primer for promoter S30
P31f	CATGAGATTTGGGAATTGACAAAGTCTTTTGTTTAGACTATAATAACGGGTTTTGTTTAACTTTA	Forward primer for promoter S31
P31r	CCCGTTATTATAGTCTAAACAAAAGACTTTGTCAATTCCCAAATCTCATGATTTCGCGGGATCGA	Reverse primer for promoter S31
P32f	CGTCTTCGAGTCACTTGACACAGTAATATACCGGAAACTATAATCGCGGGTTTTGTTTAACTTTA	Forward primer for promoter S32
P32r	CCCGCGATTATAGTTTCCGGTATATTACTGTGTCAAGTGACTCGAAGACGATTTCGCGGGATCGA	Reverse primer for promoter S32
P33f	ATTTTGACTGCTAATTGACATCGTTAATCACTGTAGGGTATAATATTACTTTTTGTTTAACTTTA	Forward primer for promoter S33
P33r	AGTAATATTATACCCTACAGTGATTAACGATGTCAATTAGCAGTCAAAATATTTCGCGGGATCGA	Reverse primer for promoter S33
P34f	CATAAGCACCGACAATTGACAAAAGACGTTAATTATGGTATAATAAATGCTTTTGTTTAACTTTA	Forward primer for promoter S34
P34r	GCATTTATTATACCATAATTAACGTCTTTTGTCAATTGTCGGTGCTTATGATTTCGCGGGATCGA	Reverse primer for promoter S34
P35f	CTTGGCAGGTCTCGGTTGACAAAAGTGCATTAGCCCCCTATAATGACCGATTTTGTTTAACTTTA	Forward primer for promoter S35
P35r	TCGGTCATTATAGGGGGCTAATGCACTTTTGTCAACCGAGACCTGCCAAGATTTCGCGGGATCGA	Reverse primer for promoter S35
P36f	TATGAAACCTTTGCTTGACACCGTATAAATCTGACCTGTATAATTGGTAGTTTTGTTTAACTTTA	Forward primer for promoter S36
P36r	CTACCAATTATACAGGTCAGATTTATACGGTGTCAAGCAAAGGTTTCATAATTTCGCGGGATCGA	Reverse primer for promoter S36
P37f	AACTGAAAAAAATTATTGACACTTGATCAATCCTAGGTATAATAGCGTAGTTTTGTTTAACTTTA	Forward primer for promoter S37
P37r	CTACGCTATTATACCTAGGATTGATCAAGTGTCAATAATTTTTTTCAGTTATTTCGCGGGATCGA	Reverse primer for promoter S37
P38f	GATTAAAAATGTTACTTGACATAAGCACTTGCAGAGGTATAATAGGATAGTTTTGTTTAACTTTA	Forward primer for promoter S38

P38r	CTATCCTATTATACCTCTGCAAGTGCTTATGTCAAGTAACATTTTTAATCATTTCGCGGGATCGA	Reverse primer for promoter S38
P39f	GCGTAGGAGCGACAATTGACAATAAAGCTGTTTGGTTTATAATAGACTCGTTTTGTTTAACTTTA	Forward primer for promoter S39
P39r	CGAGTCTATTATAAACCAAACAGCTTTATTGTCAATTGTCGCTCCTACGCATTTCGCGGGATCGA	Reverse primer for promoter S39
P40f	GCAGGCTAGTGATGATTGACAAAAGCGCGAAATTCAGTATAATAGAGAGTTTTTGTTTAACTTTA	Forward primer for promoter S40
P40r	ACTCTCTATTATACTGAATTTCGCGCTTTTGTCAATCATCACTAGCCTGCATTTCGCGGGATCGA	Reverse primer for promoter S40
P41f	TCTACTATTCGGGAATTGACAGTTTCCGTCGTGTAAGCTATAATCGCACATTTTGTTTAACTTTA	Forward primer for promoter S41
P41r	TGTGCGATTATAGCTTACACGACGGAAACTGTCAATTCCCGAATAGTAGAATTTCGCGGGATCGA	Reverse primer for promoter S41
P42f	AGATCTCCGGGCCAATTGACAATAAACTTAATTCTGTTATAATTGACTATTTTTGTTTAACTTTA	Forward primer for promoter S42
P42r	ATAGTCAATTATAACAGAATTAAGTTTATTGTCAATTGGCCCGGAGATCTATTTCGCGGGATCGA	Reverse primer for promoter S42
P43f	CTTGTTCTTAAATTTTTGACAAAAGGTGAAATTAAGGGTATAATTAACTATTTTGTTTAACTTTA	Forward primer for promoter S43
P43r	TAGTTAATTATACCCTTAATTTCACCTTTTGTCAAAAATTTAAGAACAAGATTTCGCGGGATCGA	Reverse primer for promoter S43
P44f	AATGATTCTTGTGATTGACAGGGTGACTAAGTTAGTGATATAATAGGCCTTTTTGTTTAACTTTA	Forward primer for promoter S44
P44r	AGGCCTATTATATCACTAACTTAGTCACCCTGTCAATCACAAGAATCATTATTTCGCGGGATCGA	Reverse primer for promoter S44
P45f	TATGAAACGTAGGATTGACATCCAGAGGCAAAGCCTTGTATAATGATTAGTTTTGTTTAACTTTA	Forward primer for promoter S45
P45r	CTAATCATTATACAAGGCTTTGCCTCTGGATGTCAATCCTACGTTTCATAATTTCGCGGGATCGA	Reverse primer for promoter S45
P46f	CATTGCACTTTCGGGTTGACAAAAGTGATTAAGCGTCATATAATAAGCGATTTTGTTTAACTTTA	Forward primer for promoter S46
P46r	TCGCTTATTATATGACGCTTAATCACTTTTGTCAACCCGAAAGTGCAATGATTTCGCGGGATCGA	Reverse primer for promoter S46
P47f	TGTCTCTTACTCGCTTGACATGGTTGTCTATACGCGGCTATAATAACGCC	Forward primer for promoter S47

	TTTTGTTTAACTTTA	
P47r	GGCGTTATTATAGCCGCGTATAGACAACCATGTCAAGCGAGTAAGAGAC AATTTCGCGGGATCGA	Reverse primer for promoter S47
P48f	AAAAAGCTAAACGAATTGACAGTAAGTCTTTCCTGAAGTATAATGACCC ATTTTGTTTAACTTTA	Forward primer for promoter S48
P48r	TGGGTCATTATACTTCAGGAAAGACTTACTGTCAATTCGTTTAGCTTTTT ATTTCGCGGGATCGA	Reverse primer for promoter S48
P49f	AGTTAGCAACTAACTTGACACATATGGGTTTGAGTAGGTATAATCTACGA TTTTGTTTAACTTTA	Forward primer for promoter S49
P49r	TCGTAGATTATACCTACTCAAACCCATATGTGTCAAGTTAGTTGCTAACT ATTTCGCGGGATCGA	Reverse primer for promoter S49
P50f	CAAGCGCAGGTTCTATTGACAGTTGCGCCAATCTAGTTATAATTGAGATG TTTTGTTTAACTTTA	Forward primer for promoter S50
P50r	CATCTCAATTATAACTAGATTGGCGCAACTGTCAATAGAACCTGCGCTTG ATTTCGCGGGATCGA	Reverse primer for promoter S50

*The homologous arm were underlined.

References:

1. Z. Zhang, J. Zhao, P. Wei, C. Zheng, *Comput Methods Programs Biomed* **2022**, *226*, 107087.
2. M. K. Thomason, T. Bischler, S. K. Eisenbart, K. U. Förstner, A. Zhang, A. Herbig, K. K. Nieselt, C. M. Sharma, G. Storz, *J. Bacteriol* **2014**, *197*, 18
3. W. Chen, P. Feng, H. Lin, K. Chou, *Nucleic Acids Res* **2013**, *41*, e68
4. S. Gama-Castro, H. Salgado, A. Santos-Zavaleta, D. Ledezma-Tejeida, L. Muñiz-Rascado, J. S. García-Sotelo, K. Alquicira-Hernández, I. Martínez-Flores, L. Pannier, J. A. Castro-Mondragón, A. Medina-Rivera, H. Solano-Lira, C. Bonavides-Martínez, E. Pérez-Rueda, S. Alquicira-Hernández, L. Porrón-Sotelo, A. López-Fuentes, A. Hernández-Koutoucheva, V. D. Moral-Chávez, F. Rinaldi, J. Collado-Vides, *Nucleic Acids Res* **2015**, *44*, D133.
5. Y. Chen, Z. He, Y. Men, G. Dong, S. Hu, X. Ying, *Brief. Bioinformatics* **2022**, *23*.
