# Supplementary material for: Deep Learning‐Assisted Design of Novel Promoters in Escherichia coli
Source: Adv Genet (Hoboken). 2023 Nov 15;4(4):2300184. doi: 10.1002/ggn2.202300184 (PMC10716054; doi:10.1002/ggn2.202300184)
Supplement: Supplementary file 7 — Supplementary Data S6 [file GGN2-4-2300184-s007.pdf]

ADVANCED GENETICS
Open Access

Supporting Information

for *Advanced Genetics*, DOI 10.1002/ggn2.202300184

Deep Learning-Assisted Design of Novel Promoters in *Escherichia coli*

*Xinglong Wang, Kangjie Xu, Yameng Tan, Shangyang Yu, Xinyi Zhao and Jingwen Zhou**

Supplemented File 5

Deep learning-assisted generating novel promoters in *Escherichia coli*

Xinglong Wang[1,2], Kangjie Xu[1,2], Yameng Tan[1,2], Yameng Tan[1,2], Xinyi Zhao[1,2], Jingwen Zhou[1,2,3,*]

[1] Engineering Research Center of Ministry of Education on Food Synthetic Biotechnology and School of Biotechnology, Jiangnan University, 1800 Lihu Road, Wuxi, Jiangsu 214122, China;

[2] Science Center for Future Foods, Jiangnan University, 1800 Lihu Road, Wuxi, Jiangsu 214122, China;

[3] Jiangsu Province Engineering Research Center of Food Synthetic Biotechnology, Jiangnan University, Wuxi 214122, China.

* Corresponding author: Jingwen Zhou.

Science Center for Future Foods, Jiangnan University, 1800 Lihu Road, Wuxi, Jiangsu 214122, China.

Phone: +86-510-85914371, Fax: +86-510-85914371.

E-mail: zhoujw1982@jiangnan.edu.cn.

Hyperparameters of PromoS, PromoA and PromoR

PromoS	Number of filters	Kernel size	Paddings	Stride size
Convolution Block 1	32	(1,6)	(0,5)	(1,1)
ResBlock 1	32	(1,3)	(0,1)	(1,1)
	32	(1,3)	(0,1)	(1,1)
ResBlock 2	32	(1,3)	(0,1)	(1,1)
	32	(1,3)	(0,1)	(1,1)
ResBlock 3	32	(1,3)	(0,1)	(1,1)
	32	(1,3)	(0,1)	(1,1)
ResBlock 4	32	(1,3)	(0,1)	(1,1)
	32	(1,3)	(0,1)	(1,1)
ResBlock 5	32	(1,3)	(0,1)	(1,1)
	32	(1,3)	(0,1)	(1,1)
Convolution Block 2	64	(1,2)	0	(1,2)
ResBlock 6	64	(1,3)	(0,1)	(1,1)
	64	(1,3)	(0,1)	(1,1)
ResBlock 7	64	(1,3)	(0,1)	(1,1)
	64	(1,3)	(0,1)	(1,1)
ResBlock 8	64	(1,3)	(0,1)	(1,1)
	64	(1,3)	(0,1)	(1,1)
ResBlock 9	64	(1,3)	(0,1)	(1,1)
	64	(1,3)	(0,1)	(1,1)
ResBlock 10	64	(1,3)	(0,1)	(1,1)
	64	(1,3)	(0,1)	(1,1)
Convolution Block 3	128	(1,2)	0	(1,2)
ResBlock 11	128	(1,3)	(0,1)	(1,1)
	128	(1,3)	(0,1)	(1,1)
ResBlock 12	128	(1,3)	(0,1)	(1,1)
	128	(1,3)	(0,1)	(1,1)
ResBlock 13	128	(1,3)	(0,1)	(1,1)
	128	(1,3)	(0,1)	(1,1)
ResBlock 14	128	(1,3)	(0,1)	(1,1)
	128	(1,3)	(0,1)	(1,1)
ResBlock 15	128	(1,3)	(0,1)	(1,1)
	128	(1,3)	(0,1)	(1,1)
Convolution Block 4	256	(1,2)	0	(1,2)
ResBlock 16	256	(1,3)	(0,1)	(1,1)
	256	(1,3)	(0,1)	(1,1)
ResBlock 17	256	(1,3)	(0,1)	(1,1)
	256	(1,3)	(0,1)	(1,1)
ResBlock 18	256	(1,3)	(0,1)	(1,1)
	256	(1,3)	(0,1)	(1,1)
ResBlock 19	256	(1,3)	(0,1)	(1,1)
	256	(1,3)	(0,1)	(1,1)
ResBlock 20	256	(1,3)	(0,1)	(1,1)
	256	(1,3)	(0,1)	(1,1)
Self-attention block	256			
Dense block	1			

Hyperparameters of PromoNet

PromoS	Number of filters	Kernel size	Paddings	Stride size
Convolution Block 1	32	(3,3)	0	(3,3)
Convolution Block 2	64	(1,3)	0	(1,3)
Convolution Block 3	128	(1,3)	0	(1,3)
Dense block	1			

Hyperparameters of PromoDiff

PromoS	Number of filters	Kernel size	Paddings	Stride size	MaxPool
preprocess	24	(1,6)	(0,3)	(1,1)	
	32	(1,2)	0	(1,1)	
inc	32	(1,3)	(0,1)	(1,1)	
	64	(1,3)	(0,1)	(1,1)	
Down block 1	-	-	-	-	(1,2)
	64	(1,3)	(0,1)	(1,1)	
	128	(1,3)	(0,1)	(1,1)	
Down block 2	-	-	-	-	(1,2)
	128	(1,3)	(0,1)	(1,1)	
	256	(1,3)	(0,1)	(1,1)	
Down block 3	-	-	-	-	(1,2)
	256	(1,3)	(0,1)	(1,1)	
	256	(1,3)	(0,1)	(1,1)	
Bottom block 1	256	(1,3)	(0,1)	(1,1)	
	512	(1,3)	(0,1)	(1,1)	
Bottom block 2	512	(1,3)	(0,1)	(1,1)	
	512	(1,3)	(0,1)	(1,1)	
Bottom block 3	512	(1,3)	(0,1)	(1,1)	
	256	(1,3)	(0,1)	(1,1)	
Up block 1	256	(1,3)	(0,1)	(1,1)	
	256	(1,3)	(0,1)	(1,1)	
Up block 2	128	(1,3)	(0,1)	(1,1)	
	128	(1,3)	(0,1)	(1,1)	
Up block 3	64	(1,3)	(0,1)	(1,1)	
	64	(1,3)	(0,1)	(1,1)	
Convolution Block	1	(1,1)			
